# Toward Standardized Measurement of Active Phytohemagglutinin in Common Bean, *Phaseolus vulgaris*, L.

**DOI:** 10.3390/foods14244247

**Published:** 2025-12-10

**Authors:** Henry J. Thompson, Elizabeth S. Neil, John N. McGinley, Tymofiy Lutsiv

**Affiliations:** 1 Cancer Prevention Laboratory, Colorado State University, Fort Collins, CO 80523, USA; elizabeth.neil@colostate.edu (E.S.N.); john.mcginley@colostate.edu (J.N.M.); tymofiy.lutsiv@colostate.edu (T.L.); 2 Graduate Program in Cell and Molecular Biology, Colorado State University, Fort Collins, CO 80523, USA

**Keywords:** phytohemagglutinin, lectin, common bean, pulses, ELISA, hemagglutination

## Abstract

Common bean (*Phaseolus vulgaris*, L.) is the most widely consumed grain legume globally. The seeds of common bean are a rich source of protein, but one of the seeds’ storage proteins is phytohemagglutinin (PHA), a lectin whose consumption in raw or inadequately cooked bean seed or products into which the seed is milled results in acute symptoms of food poisoning. Given that demand for incorporating common bean ingredients into foods is expanding, there has been a call for regulatory agencies to formulate more robust guidance on allowable levels of active PHA in beans and bean ingredients and for establishing standardized methodology for measuring active PHA. Herein, detailed protocols are provided for extraction of PHA from beans and for the use of digital image analysis in the traditional hemagglutination assay. Results are compared to an ELISA assay. Given reports that ingestion of four to five soaked raw dark red kidney bean (DRK) seeds can induce food poisoning, our focus was on this market class of bean. By ELISA assay, estimated concentration of active lectin in DRK was 223 ± 0.07 mg/g dry weight and the total amount of PHA contained in four seeds was 544 mg. Commercially cooked canned beans had >99% reduction in PHA (4.9 µg/g dry weight). Consumption of an entire can (1.5 cups, equivalent to 94 g dry matter) would equal 0.46 mg PHA which is approximately 1000-fold lower than the amount estimated to be associated with food poisoning. It is hoped that this report stimulates continued interest in standardizing methodology across laboratories and in setting standards of identity for active PHA in bean products.

## 1. Introduction

Grain legumes, which are also termed pulses, are a rich source of dietary protein. Of the four commonly produced beans, i.e., common (dry) bean, chickpea, dry pea, and lentil, the most widely consumed pulse globally is common bean, which is the focus of this investigation. Among the many protein types stored in bean seed [1,2], the most investigated is phytohemagglutinin (PHA) (≥10% *w*/*w*), a lectin [3]. Plant lectins are glycoproteins containing carbohydrate recognition sites, typically involved in plant defense against various pests, including microbes and different types of insects [4]. If bean seed or the flours into which it is milled are not adequately heat-treated to inactivate PHA, prior to consumption, active PHA in the bean can induce acute food poisoning, i.e., nausea, vomiting, diarrhea, and intestinal discomfort within 1 to 3 h of ingestion [5,6]. The most significant number of reports of accidental food poisoning are for the dark red kidney bean market class, which is among those investigated herein [7]. Given consumer interest in plant-based protein, and the potential for increased consumption of beans and for incorporating bean flours into various processed foods [8], there was a recent call for harmonizing the methodology for measuring active PHA content across laboratories and governmental or private sector agencies in order to assist with product development and to monitor food safety [7]. The goal is to accurately assess the exposure dose of active PHA, which requires multiplying the concentration in the food by the typical amount of the food that will be consumed. This is the basis for risk assessment in determining the safety of foods relative to PHA.

Available methods used to determine PHA activity include affinity chromatography, immunoenzymatic assays, e.g., ELISA, mass spectrophotometry, and hemagglutination [9,10,11,12]. The hemagglutination assay is the most widely used method for assessing the lectin activity of raw, cooked, or processed plant food products [7]. While the assay is rapid and economical, it has limited specificity, is prone to dilution errors, and uses a subjective visual determination of the agglutination endpoint, and the reporting unit, HAU, is challenging to use in determining the concentration (mg PHA/g food) of active PHA present in a food [13]. Because it is commonly used, the work reported herein focused on improving the objectivity of the hemagglutination assay endpoint determination and estimating the amount of PHA detectable when the assay endpoint (lack of agglutination) is attained. In addition, because of the physical hardness of bean and particles within the flours into which it is milled, the procedure for extracting raw seed and flours was evaluated to maximize extraction efficiency. In addition, the results of exposure dose computation using an ELISA assay are contrasted with those from the hemagglutination assay.

## 2. Materials and Methods

### 2.1. Reagents

#### 2.1.1. Hemagglutination Reagents

Salt-free phytohemagglutinin (PHA-P) from Phaseolus vulgaris (red kidney bean) was obtained (cat. L8754, Millipore-Sigma, St. Louis, MO, USA). Note: the purity of this PHA-P is usually 60–70%, e.g., a weight of 1.6 mg/mL will likely result in protein concentration of approximately 1 mg/mL as measured by BCA. BCA protein determination kit (cat. 23227 Pierce BCA Protein Assay Kit, Thermo Scientific, Waltham, MA, USA). Rabbit defibrinated whole blood (cat. DRB030, Hemostat Laboratories, Dixon, CA, USA). Phosphate-buffered saline (PBS) 1 L volume: 8 g sodium chloride, 0.2 g potassium chloride, 1.44 g sodium phosphate dibasic, 0.24 g potassium phosphate monobasic, adjust PBS to pH 7.4.

#### 2.1.2. ELISA Reagents

PBS pH 7.4, PBS with 0.05% Tween 20 pH 7.4 (PBST), 50 mM (0.05 M) Carbonate-Bicarbonate Buffer pH 9.8 (CBB) 1 L volume: 2.049 g sodium bicarbonate, 2.26 g sodium carbonate anhydrous. Bovine serum albumin (BSA) protease-free, globulin-free (cat. A7030, Millipore-Sigma, St. Louis, MO, USA). PBS with 0.5% BSA (PBSB) was used for blocking, a minimum of 13.5 mL/plate. PBS with 0.25% BSA (PBSB) was used for antibody dilutions, a minimum of 27 mL/plate. Porcine Thyroglobulin 8 µg/mL (cat. T1126, Millipore-Sigma, St. Louis, MO, USA) or Fetuin 8 µg/mL (cat. F3004, Millipore-Sigma, St. Louis, MO, USA). Standards: Phytohemagglutinin (PHA-P, salt-free, cat. L8754 Millipore-Sigma, St. Louis, MO, USA), lectin from Phaseolus vulgaris-red kidney bean 1 mg/mL (concentration confirmed by BCA). Primary Antibody: Rabbit-Anti-PHA-P (E + L), (cat. AS-2300-1, Vector Laboratories, Newark, CA, USA), diluted to 4 µg/mL in 0.25% BSA (PBSB). Secondary Antibody: mouse anti-rabbit IgG conjugated to horseradish peroxidase (cat. 211-035-109, Jackson Immuno Research, West Grove, PA, USA). Note: dilution of the secondary changes according to coating of the plate (use a 1:500 intermediate dilution to make the final dilution). For plates coated with fetuin, the secondary antibody was diluted to 1:50,000. For plates coated with thyroglobulin, the secondary antibody was diluted to 1:20,000. Substrate: O-phenylenediamine dihydrochloride (OPD, cat. 34005, Thermo Fisher Scientific, Waltham, MA, USA), stable peroxide buffer (cat. 34062, Thermo Fisher Scientific, Waltham, MA, USA). 2.5 M sulfuric acid was used to stop the colorimetric reaction at 30 min.

#### 2.1.3. PAGE Reagents

Purified PHA was purchased from two commercial vendors: Millipore-Sigma and Vector Laboratories. The following types of PHA in addition to raw and cooked dark red kidney (DRK) bean seed, 10% (*w*/*v*), were evaluated: PHA-P (salt-free, cat. L8754, Millipore-Sigma, St. Louis, MO, USA); PHA-P (salt, cat. L1668, Millipore-Sigma, St. Louis, MO, USA); PHA-E (cat. L8629, Millipore-Sigma); PHA-L (cat. L2769, Millipore-Sigma); PHA-M (cat. L8902, Millipore-Sigma); PHA-E (cat. L-1120-5, Vector Laboratories, Newark, CA, USA); PHA-L (cat. L-1110-5, Vector Laboratories, Newark, CA, USA); PHA sources are listed (Supplementary Table S1).

### 2.2. Sample Preparation

*Raw bean seed.* Whole bean seeds for several popular market classes were obtained from a retail grocery store (Sprouts Farmer’s Market, Inc., Phoenix, AZ, USA and The Kroger Co., Cincinnati, OH, USA) to mirror beans to which a consumer would have access. Bean seed was placed into a 3-oz. coffee grinder (Krups, Solingen, Germany) and ground to a fine powder using a series of repeated 5 s pulses. Ground powder was stored in 15 mL conical tubes and frozen at −20 °C.

*Commercially canned beans.* The content of each can of commercially available bean was weighed and poured into a clean Ninja Smoothie cup (model SS101, SharkNinja, Needham, MA, USA). The contents were homogenized using the extract mode for 30 s. The homogenized bean seed was poured into a gallon Ziploc bag; the cup and blades were rinsed with deionized water and poured into the same bag. The bag was sealed and placed flat at −70 °C until frozen, at which point the frozen contents of a bag were loaded onto separate aluminum foil-lined trays, placed into a freeze dryer (Harvest Right, Salt Lake City, UT, USA), and freeze-dried under vacuum for 24 h. The freeze-dried material was ground to a fine powder in a coffee grinder and stored in 15 mL conical tubes at −20 °C until ready for use.

### 2.3. Sample Extract Homogenization and Centrifugation

Sample extraction was optimized for type of beads, number of beads per tube, number of cycles and speed of the Bead Ruptor Elite homogenizer, and dwell time between cycles. The final protocol was as follows: ten 2.3 mm yttria-stabilized zirconia ceramic beads (cat. 11079123zxy, Biospec Products, Bartlesville, OK, USA) were placed in each bead-beating tube (XXTuff2, cat. 330TX821, Biospec Products). Tubes were tared and approximately 150 mg of sample powder was added to each tube and then PBS, pH 7.4 was added to create a 10% (*w*/*v*) extract. Sample tubes were placed on ice for 1 min prior to loading on a Bead Ruptor Elite (Revvity-Omni International, Kennesaw, GA, USA) bead mill homogenizer. Tubes were run for two cycles at a speed of 6 m/s for 30 s with a 1 min dwell time on ice between cycles. Tubes were centrifuged at 15,000× *g* for 15 min at room temperature. Supernatant was transferred to a clean 2.0 mL tube and stored frozen at −20 °C. Sample tubes were thawed at room temperature prior to use.

During protocol optimization, a small portion of the centrifuged pellet from both the vortexed and bead-mill-homogenized samples was resuspended in 1 mL of PBS. Then, 35 µL of Lugol’s iodine was added, briefly vortexed, and a 50 µL droplet of the mixture was placed on a microscope slide using a single-channel pipette. Images were captured using a customized system consisting of a 3.0-megapixel CMOS camera (Clemex Technologies, Inc. Longueuil, QC, Canada) attached to a Z16 APO monocular zoom lens (Leica Microsystems, Inc., Bannockburn, IL, USA). The camera and lens were attached to a Z motor (Leica Microsystems, Inc.) above a transmitted light base with a 100 × 100 mm motorized stage (Clemex Technologies, Inc.). A joystick attached to an X–Y control box (Clemex Technologies, Inc.) was used to move from one image field to the following field on the slide. Images were captured at 92× magnification using Captiva v4.0 software (Clemex Technologies, Inc.) and saved as JPEG files.

### 2.4. Polyacrylamide Gel Electrophoresis (PAGE) to Assess Appropriate PHA Control

For native polyacrylamide gels, sample extracts were diluted with 20 µL of 2X sample buffer consisting of 62.5 mM Tris-HCl (pH 6.8), 25% glycerol, and 0.01% bromophenol blue. Sample extracts for denatured gels were diluted using 2X sample buffer containing the same reagents listed above, with the addition of 2% sodium dodecyl sulfate (SDS) and 2 µL of 2-mercaptoethanol. Protein concentration previously determined by BCA was used to gauge proper dilution with equal protein concentration loaded across all wells in each gel. Milli-Q water was added to both native and denatured mixtures, bringing the final volume to 30 µL with a final sample protein concentration of 0.10 µg/µL. Sample tubes containing SDS were heat-denatured in a dry heat block at 85 °C for 2 min, cooled to room temperature, and stored on ice until loaded into the gel.

Precast 12-well Invitrogen Novex Tris-Glycine Mini Gels cassettes (cat. XP04202BOX, Thermo Fisher Scientific, Waltham, MA, USA) were used in conjunction with a XCell SureLock Mini-Cell Gel Rig (cat. EI0001, Thermo Fisher Scientific, Waltham, MA, USA) with Novex power supply adapters connected to a power supply (Bio-Rad, Hercules, CA, USA). Combs were removed from the precast gel cassettes, and the wells were filled with 1X running buffer and then drained. This process was repeated twice more to ensure that all wells were properly rinsed with running buffer. The gel cassette was loaded into the chamber and locked into position before the chamber was filled with running buffer.

Samples were loaded into wells 2–11 using gel-loading tips, with a total volume of 30 µL per well. Protein ladders were loaded on both sides of the gel (wells 1 and 12), 5 µL in each well. The pre-stained PageRuler Plus 10–250 kDa protein ladder (cat. 26619, Thermo Fisher Scientific, Waltham, MA, USA) was used for the denatured gel and was not heat-denatured prior to loading. An unstained protein ladder was used for the native gel, NativeMark 20–122 kDa (cat. LC0725, Thermo Fisher Scientific, Waltham, MA, USA).

**Figure 1:**
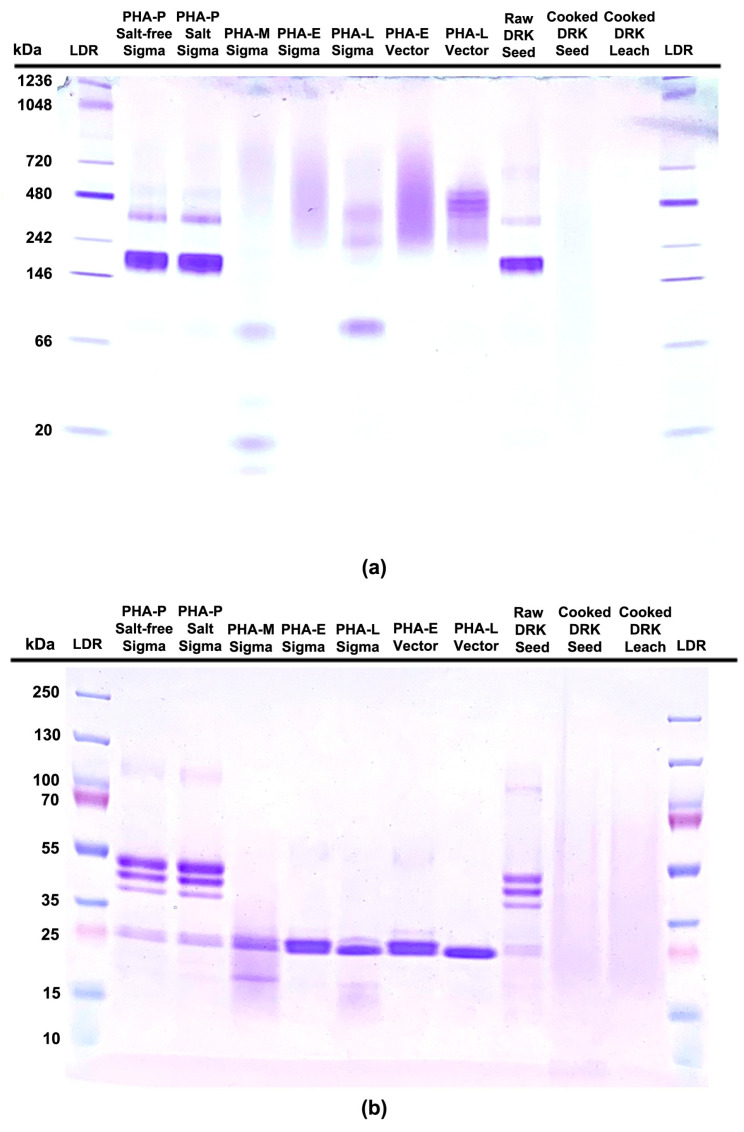
Polyacrylamide gel electrophoresis (PAGE): (**a**) Native gel depicting different forms of PHA from two different vendors along with raw and cooked dark red kidney bean seed and cooked DRK leachate; (**b**) SDS PAGE showing the same samples as listed in the native gel, but in the denatured form; DRK: dark red kidney; LDR: ladder; PHA: phytohemagglutinin. Original gels post-electrophoresis were physically pitched inward at the top. Gel images were straightened, and exposure was increased for clarity. However, the original gel images are shown in the Supplementary Materials (Supplementary Figure S1).

Gels were run at 120 V for 1 h. 15 min for the denaturing SDS gel and at 120 V for 1 h. and 20 min for the native gel. The plastic cassettes were removed from the chambers, opened, and the top loading wells were cut off, while the top corner of the gel was notched using a gel knife to indicate the orientation of the starting well. Gels were submerged in deionized water for 5 min to allow the gel to detach from the cassette. Once released, gels were rinsed in deionized water three times prior to staining with 0.1% Coomassie Brilliant Blue R250 in 50% methanol with 7.5% glacial acetic acid for 30 min at room temperature on an orbital shaker. Stain was decanted and rinsed in running deionized water until clear. Water was drained away from the gel, approximately 100 mL of destaining solution (10% methanol, 7.5% glacial acetic acid, and 82.5% deionized water) was added to the container, and the gel was gently mixed for approximately 2 h on the orbital shaker until the desired background was achieved. The destaining solution was decanted, the gels were rinsed in deionized water, transferred to a clear plastic tray with deionized water, placed on a lightbox, and photographed (Figure 1).

### 2.5. Hemagglutination Assay

The assay was performed on a standard 96-well clear round-bottom plate. PBS (pH 7.4) was added to wells 2–12 using a multichannel pipette with a reverse pipetting technique, 100 µL per well. The PHA-P positive control was loaded into the first well of the first row (A1) using a single-channel pipette, 200 µL in the well. Samples were loaded into the first well of each subsequent row (B1–G1) using a single-channel pipette, 200 µL in each well. Lastly, PBS (pH 7.4) was loaded into the first well of the last row (H1) in the plate (200 µL) and used as a negative control. The PHA-P control, samples, and negative control were serially diluted using a multichannel pipette with the forward pipetting technique: 100 µL were removed from the first column of wells, transferred to the second column, and mixed 10 times. This process was repeated for each subsequent column of wells until reaching the end of the plate, where 100 µL was withdrawn from the 12th column and discarded, leaving a 100 µL volume across all occupied wells. We found that cooked or suspected low-lectin-containing samples, in general, required only a single plate with 12 wells for serial dilutions. If necessary, for raw samples or suspected high-lectin-containing samples, as was the case with raw dark red kidney (DRK) bean, serial dilutions were extended into a second plate for a total of 24 wells. Suggested plate layouts are provided (Supplementary Figures S2 and S3).

Defibrinated rabbit red blood cells (RBCs) solution (cat. DRB030, Hemostat Labs, Dixon, CA, USA) was diluted in PBS (pH 7.4) to 2.5% and gently mixed by inverting the 15 mL conical tube a few times. The diluted RBCs were added to each well of the plate(s) using a multichannel pipette with a reverse-pipetting technique (100 µL per well), resulting in a total volume (serially diluted sample and RBCs) of 200 µL per well. Plates were shaken using a titer plate shaker (Labline Instruments, Inc., Melrose Park, IL, USA) at speed 3 for 30 s. Any visible air bubbles in the wells were popped using a 25 G needle. Plates were covered and allowed to sit undisturbed at room temperature prior to photographing at 2 h and again after overnight incubation.

Plates were photographed using the camera on an iPhone 16 (Apple, Cupertino, CA, USA) with 2X zoom attached to a tripod over a backlit lightbox with a white diffusing panel, e.g., porta-trace lightbox (Gagne, Inc., Johnson City, NY, USA). Both grid and level options were enabled in the camera app to aid proper alignment and leveling of the iPhone before image capture. To minimize parallax, the camera height on the tripod was kept at 75 cm above the lightbox [14]. Images were focused by gently tapping the iPhone screen over the central portion of the plate before capturing the image. Images were rotated, cropped, and exposure increased by 30 points using the camera app prior to importing images to a desktop computer. Hemagglutination plates incubated overnight show greater clarity than those incubated for only 2 h, making the subjective process of delineating the last positive and first negative wells by eye easier than it would otherwise be.

### 2.6. Image Analysis

Given the highly subjective nature of visually distinguishing positive from negative hemagglutination results using the human eye as commonly practiced, our lab chose to use image analysis to provide a more objective and reproducible approach. Captured images from hemagglutination plates incubated overnight were analyzed using an image analysis macro developed by our laboratory. Briefly, color plate images were imported into the open-source ImageJ (ver. 1.5.4) image analysis software (https://imagej.net). The scale was set by measuring the distance from edge to edge of one well in the image using the line tool, and by using the plate manufacturer’s known well bottom diameter. Measurements for area and centroid (X, Y location) were also set. Images were rotated slightly to better align the rows of wells, cropped to the outer edges of the plate, and then converted to 8-bit grayscale.

A region of interest (ROI) overlay was drawn on the image using the oval tool, covering slightly less than the total inner area of one well, starting in the upper-left corner of the plate. The initial ROI was duplicated eleven times using the ROI manager, moving each new ROI to the adjacent well to the right, creating a total of 12 ROIs directly superimposed over the 12 wells in the first row of the plate (A). The 12 individual ROIs were combined into a single ROI that could be moved between rows. A loop began by prompting the user to analyze a row of wells. If yes, the macro continued by creating a binary mask of the combined ROI, which was used to extract the 12-well bottom ROIs from the first row of 12 wells in the plate into a new image with a white background. The threshold range was set at 0–235 out of 256 possible shades of gray.

The “Analyze Particles” function was used to measure the area and centroid of each well bottom. Values were written to the results table in ImageJ and sorted by centroid, with the smallest X values (horizontal) at the top and largest at the bottom, which aligned with the left-to-right position of the 12 wells in a plate row. The results table data was copied to the clipboard, and the user was prompted to paste the data into a Microsoft Excel file. The user was then prompted to analyze a row of wells. If yes, the macro repeated the loop, allowing the user to move the combined ROI to the next row of wells before analysis; if not, the macro halted execution.

Data were graphed in Microsoft Excel with well number on the X axis vs. area (mm^2^) on the Y axis. Negative controls (PBS and RBCs) must be included along with a PHA-P positive control and samples when performing image analysis. Negative controls are crucial for establishing the mean reference line on the graph where samples transition from positive to negative. We defined a positive hemagglutination area value as three standard deviations above the mean negative area. An edge effect was observed in the first few serial dilutions of some higher lectin-containing samples, resulting in small polygonal hemagglutinated well areas and lower square millimeter area measurements compared to higher serial dilutions, which showed a larger area of hemagglutination before ultimately decreasing to a true negative well. This edge effect is an artifact of the hemagglutination assay and, therefore, should be ignored when looking at area measurements on the graph to determine the last positive well vs. first negative well (Supplementary Figure S8). The ImageJ macro for image analysis of hemagglutination plates is freely available for use (https://github.com/mcginleyj/hemagglutination (accessed on 25 November 2025). Negative controls should be run with each hemagglutination assay, as slight drift in area measurements can occur due to the age of the RBCs.

### 2.7. ELISA Assay

Briefly, the ELISA assay was adapted from previous reports [3,13]. The assays were optimized using a primary antibody concentration of 4 µg/mL in PBS, pH 7.4 with 0.25% BSA. Plates were coated with thyroglobulin or fetuin at 8 µg/mL. The concentration of the secondary antibody was also optimized to 1:20,000 for thyroglobulin-coated plates and 1:50,000 for fetuin-coated plates in PBS, pH 7.4, with 0.25% BSA (PBSB). O-phenylenediamine Dihydrochloride (OPD) diluted in ultrapure water, 0.5 mg/mL, with 10× stable peroxide buffer was used as the substrate. The reaction was stopped at 30 min by adding 50 µL of 2.5 M sulfuric acid to each well, and the plate was read at 490 nm. A detailed protocol is presented (Supplementary PHA-P ELISA Protocol S2).

## 3. Results

### 3.1. PHA Standard

Recognizing the importance of a defined and widely available PHA standard for assay calibration within and across laboratories, various types of PHA were obtained from two commercial vendors, Millipore-Sigma and Vector Laboratories (Supplementary Table S1). From published work on common bean in which PHA has been evaluated, PHA-P and PHA-M are most often cited, although PHA-E and PHA-L have also been used [3,7,11,12]. To create a basis for reagent selection, these protein preparations were run on native and denaturing PAGE gels and compared to raw and cooked DRK bean extracts (Figure 1). PHA-P showed an electrophoretic profile very similar to that of raw DRK bean seed and, therefore, was chosen as the PHA standard reagent used for the remainder of the work reported herein.

### 3.2. Extraction and Centrifugation

**Figure 2:**
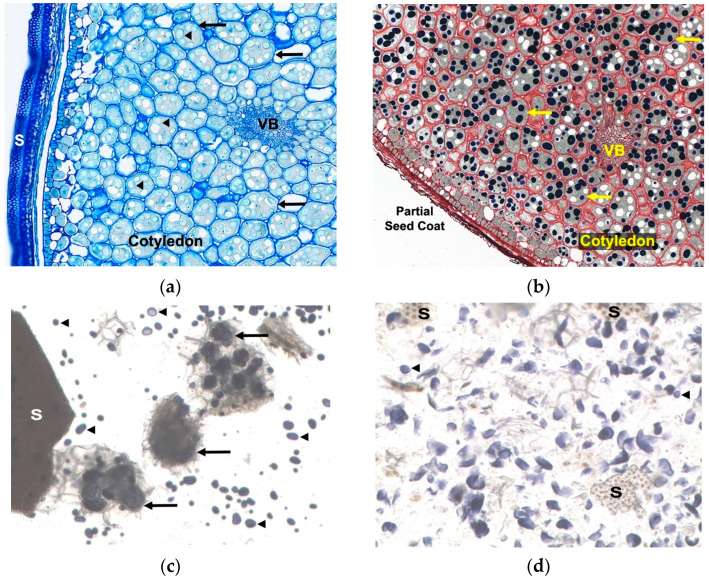
Dark red kidney (DRK) bean seed histology: (**a**) 10 µm cross section of formaldehyde alcohol acetic acid (FFA) fixed paraffin embedded DRK bean seed stained with toluidine blue O for 1 min, dehydrated and mounted with synthetic resin, parenchyma cells (arrows), starch granules (arrowheads), seed coat (S), vascular bundle (VB) are indicated; (**b**) 10 µm cross section of FFA fixed paraffin embedded DRK bean seed stained with toluidine blue O for 1 min followed by Lugol’s iodine for 30 s, aqueous mount, parenchyma cells (arrows), iodine stained starch granules appear as dark blue to black spheres, white vacuoles are missing starch granules due to cutting artifact, partial seed coat and vascular bundle (VB) are indicated; (**c**) Ground raw DRK bean powder prepared with PBS, pH 7.4, 10% (*w*/*v*) extract, placed on a multitube vortexer for at speed 6 out of 10 for 60 min, centrifuged at 15,000× *g* for 15 min, pellet resuspended in PBS and stained with Lugol’s iodine and mounted with aqueous medium, intact aggregates of parenchyma cells (arrows), liberated intact starch granules (arrowheads), intact seed coat (S) are indicated; (**d**) Ground raw DRK bean powder prepared with PBS, pH 7.4, 10% (*w*/*v*) extract, placed in a bead mill homogenizer with thirty 2.3 mm stainless steel beads and sample tubes homogenized at a speed of 6 m/s for 10 cycles of 30 s with 30 s dwell time between cycles in ice cold acetone, centrifuged at 15,000× *g* for 15 min, pellet resuspended in PBS and stained with Lugol’s iodine, aqueous mount, a few intact starch granules (arrowheads) are present while the majority of granules appear fragmented, thin layer of residual seed coat (S) is present.

The ground powder of bean seed is recognized to contain hard particles that are challenging to homogenize, and PHA is stored in vacuoles within the seed, and vacuoles can be difficult to disrupt [15]. In an effort to extract as much PHA as possible from bean samples, we evaluated using a bead mill homogenizer as described in Section 2.3 compared to vortexing alone, the most common method reported in the literature [3,13,16,17,18]. Vacuoles containing starch were used as a surrogate for lectin-containing vacuoles because their detection is simpler. Images of stained vortexed and bead mill homogenized samples are shown along with toluidine blue O and Lugol’s iodine-stained cross sections of fixed DRK bean seed for comparison (Figure 2). Stainless-steel beads imparted a very noticeable gray discoloration of the homogenized samples in the tubes. Therefore, we proceeded using only ceramic beads, which did not alter the color of the homogenized samples. Subsequent tests revealed that, as recommended by the manufacturer, 30 beads were unnecessary and that 10 ceramic beads per tube were sufficient. Moreover, reducing the number of cycles from ten to two 30 s cycles still provided adequate sample homogenization. Incubating sample tubes in an ice-cold acetone bath between cycles was too cold, resulting in a completely frozen mixture of beads and sample within the tubes. Use of ice alone was found to be sufficient for cooling samples between homogenization cycles.

Preliminary testing was performed using the FastPrep-24 instrument. However, we found that removing sample tubes from this instrument for incubation on ice between homogenization cycles was cumbersome and time-consuming. In addition, the lid lock mechanism, which must be engaged for operation, was challenging to use, requiring the user to push the lid down with one hand while engaging the mechanical latch on the side of the instrument with the other. In contrast, the Bead Ruptor Elite instrument provided sample homogenization equivalent to the FastPrep-24; the front-facing, angled tube adapter design reduced loading/unloading times, and the instrument featured a more ergonomic magnetic lid locking mechanism that could be easily operated with one hand.

In order to quantify differences in extraction efficiency across methods, total protein content of the supernatant fraction was determined (Table 1). The superiority of the Bead Ruptor Elite is clearly demonstrated.

#### Effect of Centrifugation Speed on Clarity of Sample Extracts

Based on a review of literature, centrifugation speed and times for preparation of sample extracts is highly variable, e.g., 350–10,000× *g* for 3–35 min [3,13]. We tested the following centrifugation speeds and times: 1000× *g*, 7500 × *g*, 15,000× *g*, and 20,000× *g* for 3, 8, 15, and 20 min, respectively, at 4 °C and room temperature. We found that a higher speed for a more extended period, e.g., 15,000× *g* for 15 min, yielded a consistently visually clear supernatant. Clear supernatants are vital for all assays that require optical clarity, e.g., BCA, hemagglutination, ELISA. Lower centrifugation speed resulted in significant particulates in the sample extracts, making it very difficult to determine correct calls of positive versus negative wells visually in the hemagglutination assay, especially when evaluating cooked sample extracts (Supplementary Figures S4 and S5).

### 3.3. Analysis of PHA

#### 3.3.1. Hemagglutination of Purified PHA-P

**Figure 3:**
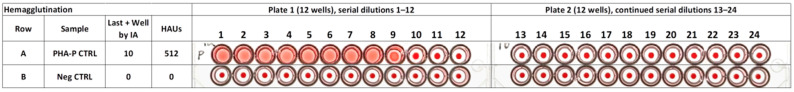
Hemagglutination of PHA-P positive control. The last positive dilution well for hemagglutination was determined by image analysis. PHA-P control with starting concentration of 1.04 mg/mL as measured by BCA; HAU: hemagglutination unit; IA: image analysis; Neg CTRL: negative control; PHA-P: type of phytohemagglutinin.

Red blood cells (RBC) agglutinated by the presence of active PHA-P lectin will be displayed as a mat across the surface of the well. In addition, the edges of the mat may roll up onto itself, creating an edge effect with variable-shaped polygons on the surface of the well, especially at higher concentrations of sample. The rollup “phenotype” is generally not observed with purified PHA-P, but is commonly observed with bean extracts (Supplementary Figure S4). As samples are diluted farther out, the edge effect subsides, and the mat spreads out to cover all or most of the well. Further dilution will cause the mat to become smaller, fuzzier, and more oblong, and in some cases, a donut hole may appear in the center. Eventually, the sample will become so diluted that agglutination is no longer possible and the individual RBCs fall to the bottom of the well, congregating at the center in a crisp, well-formed red dot, resulting in the first negative well with all subsequent serial dilutions appearing the same. The last positive well is the well prior to the first negative well, e.g., PHA-P at a starting concentration near 1 mg/mL will typically be negative in the 11th and 12th wells, and slightly positive in the 10th well at a dilution of 1:512 with a reported HAU value of 512. The 10th well contains 203 ng of PHA-P (Table S2 and Figure 3). A detailed step-by-step hemagglutination assay protocol is available (Supplementary Protocol S1).

#### 3.3.2. Estimated Amount of PHA-P Detectable by Hemagglutination Using the Digital Image Analysis Algorithm to Detect the Last Positive and First Negative Well

**Figure 4:**
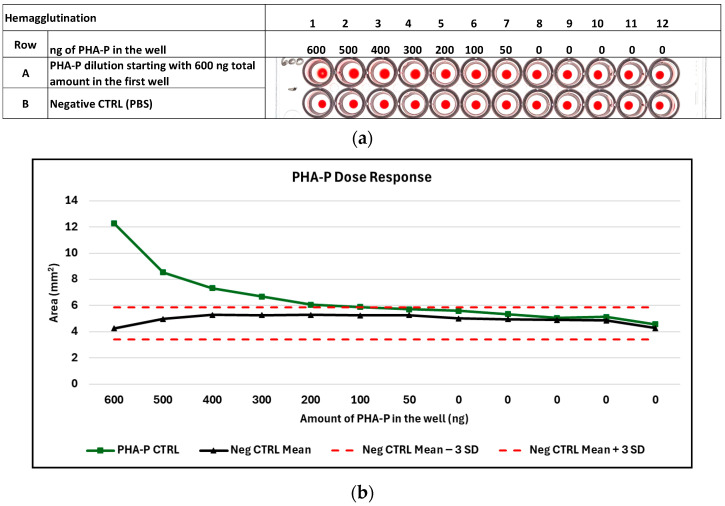
Image analysis of PHA-P dose response: (**a**) Hemagglutination results of PHA-P positive control starting with a 600 ng amount in the well and diluting in 100 ng increments with a final dilution of 50 ng in the well compared to the negative control; (**b**) Graph of the PHA-P dose response showing a stepwise reduction in hemagglutination area (mm^2^) measurements by digital image analysis with a minimal detectable amount of PHA-P at 200 ng; Neg CTRL: negative control; PHA-P: type of phytohemagglutinin.

As serial dilutions are carried out, the dilution range between adjacent wells becomes larger, and the transition from lectin positive to negative is the difference of one well, with the minimum amount of lectin detected falling somewhere between the two wells. To investigate the minimal amount of PHA-P detectable in the hemagglutination assay, we started with 600 ng, an amount of PHA-P present between serially diluted wells 8 and 9 in the assay, based on a starting amount concentration of 1.04 mg/mL (104,000 ng; Table S2). Dilutions stepped down from 600 ng to 100 ng in increments of 100 ng with a final dilution of 50 ng in a well. The minimal amount of PHA-P detectable by image analysis in the hemagglutination assay was 200 ng (Figure 4).

#### 3.3.3. Hemagglutination of Raw DRK Bean Seed

**Figure 5:**
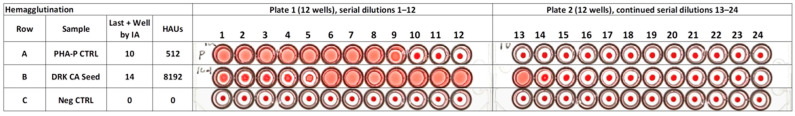
Hemagglutination results of a commercially available raw DRK bean seed using image analysis; CA: commercially available; DRK: dark red kidney; HAU: hemagglutination unit; IA: image analysis; Neg CTRL: negative control; PHA-P CTRL: phytohemagglutinin isoform P positive control.

**Figure 6:**
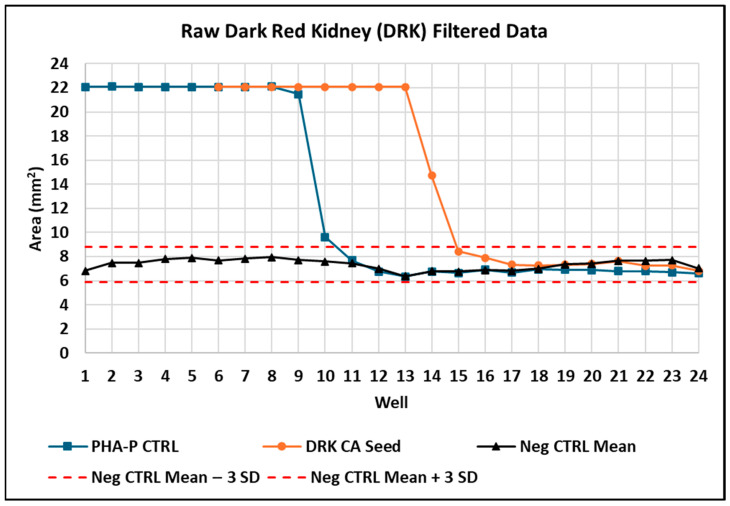
Image analysis profile of a commercially available raw DRK bean seed. Image-derived data analysis of hemagglutination results (artifact removed via filtering); hemagglutination area (mm^2^) values > 3 SD above the mean negative control indicate active lectin in a well; CA: commercially available product; DRK: dark red kidney; HAU: hemagglutination unit; IA: image analysis; Neg CTRL: negative control; PHA-P CTRL: phytohemagglutinin isoform P positive control.

We selected DRK bean seed from a commercially available retail source and found its active PHA with a last positive dilution well of 14 (8192 HAUs), corresponding to 1220.7 ng of sample powder in the well (Table S4). Hemagglutination plate results are shown (Figure 5) along with corresponding graphed image analysis results (Figure 6). The raw unfiltered image analysis graph is also shown (Supplementary Figure S8). A raw DRK bean seed containing a high amount of PHA shows edge-effect artifacts clearly visible in wells 1–5 (Figure 5, row B).

The estimated amount of active PHA that induces detectable agglutination is 200 ng. Loss of agglutination is observed between wells 14 and 15 (Figure 6). There are 1221 ng of bean powder extract in serial dilution well 14 and 610 ng in serial dilution well 15 (Supplementary Table S4). It is common for the loss of agglutinating activity to fall between two serial dilutions. In this example, the estimated amount of PHA in DRK bean was 327 mg/g dry bean, i.e., 200 ng PHA divided by the estimated 610 ng bean powder in the well.

### 3.4. ELISA

Based on literature reports that the lower limit of active PHA by ELISA is 15 ng [13], a standard curve for the ELISA assay using PHA-P with the following amounts in ng: 0, 12.5, 25, 50, 100, 200 and 300 (Supplementary Figures S9 and S10). The regression coefficients for the standard curves generated using fetuin or thyroglobulin were 0.0039 (95% CI: 0.0037–0.0041) absorbance units per ng PHA and 0.0020 (95% CI: 0.0019–0.0021) absorbance units per ng PHA, respectively. The results presented here are the fetuin-generated data. Hemagglutination results by image analysis were used as a guide to identify the initial dilution factor for each bean market class to be analyzed by ELISA. Two dilutions were evaluated, for example, with DRK 1:8192 and 1:16384, with the goal of detecting absorbance values close to 1.0.

The estimated amount of PHA determined by ELISA is shown (Table 2). It is important to note that the dry weight is estimated based on the actual dry weight of the ground bean seed that was extracted (10% *w*/*v*). The dark red kidney bean and white kidney bean market classes were selected because they are most frequently reported sources of beans in accidental cases of food poisoning. Black bean and pinto bean were selected as they are commonly consumed market classes. Three different pinto beans were evaluated to examine application of the ELISA assay across a board range of PHA concentrations. Heidi pinto beans were kindly provided to us, as a PHA null variety to use in assay validation. PNT 2 is actually the Monterrey pinto cultivar that we determined was a low PHA cultivar during work on making bean bread. PNT 1 was from a commercial retail source.

To further examine the usefulness of the ELISA, it was used to evaluate the concentration of active PHA in the same market classes of commercially canned beans. Interest in this analysis was sparked by a report of active lectin in canned beans using the hemagglutination assay, but there was no effort in that paper to relate the concentrations reported in HAU units to an exposure dose that induces food poisoning [19]. Canned beans (Table 3) have less than 1% of the PHA reported in raw beans (Table 2).

## 4. Discussion

### 4.1. Overview

The goal of assessing the active PHA content of common bean and bean flour fractions is to provide guidance to the food industry during food product development and to ensure the safety of foods relative to PHA-induced food poisoning. There are acknowledgments by regulatory groups in several countries of the risks posed to consumers due to the lack of standards of identity for PHA and the absence of food product monitoring [7,13,20]. To promote increased consumption of common bean while maintaining consumer confidence, the safety of food is of utmost importance. In looking toward method standardization, we decided to examine sample extraction and improve the objectivity of the hemagglutination assay, since some labs are unlikely to abandon the approach and replace it with an ELISA. In the sections below, we briefly summarize key findings and then provide useful insights from the application of the method development work reported.

### 4.2. Sample Extraction

In the multi-omics world, extraction efficiency is a key consideration, and it is generally recognized that achieving high, consistent extraction efficiency requires homogenization with a bead mill homogenizer, using beads of specific sizes and made from various materials, depending on the type of extraction. Generally, in the extraction of PHA from bean into PBS from a ground powder, the sample is agitated using a vertical vortex mixer. The problem we recognized was that washing the extracted pellet up to eight times still revealed active PHA, as detected by the hemagglutination assay. While such an approach may be sufficient for qualitative comparisons among different samples of similar composition, safety testing demands high extraction efficiency. From the perspective of plant biology, these findings were also consistent with the recognized fact that beans and other pulses store the majority of the lectins they produce in vacuoles, which can be difficult to lyse [15,21]. Of the many approaches that were assessed, bead mill homogenization was deemed the method of choice, and our approach is summarized in Section 2.2. The other component of the extraction process that warrants comment is the centrifugation of the extract after bead-mill homogenization. Most protocols that use a vortex mixer to extract in PBS report using low-speed centrifugation, e.g., 1000× *g* for 3 min. Our experience was that supernatants prepared in this manner still contained a significant amount of particulates, which are not conducive for downstream applications. PHA will not come out of solution at higher g forces. In our hands, 15,000× *g* for 15 min yielded a consistently clear supernatant, which facilitated the determination of PHA using either the hemagglutination or ELISA assay.

### 4.3. Positive Control/Standard: Selection and Preparation

Commercially available purified types of PHA (M, E, L & P) were obtained and evaluated by PAGE (Section 2.4 and Section 3.1, Figure 1). PHA-P had a profile most similar to that of extracts from raw DRK bean seed and was selected as a positive control, which is run on every hemagglutination plate and used for quality control (QC) to monitor plate acceptability and detect assay drift. It is absolutely critical when making stock solutions of PHA-P that an independent protein analysis be performed to confirm the concentration of PHA-P in each batch of stock PHA-P. The BCA assay is highly suitable for this purpose. PHA-P is also used to generate a standard curve for each ELISA plate, where it is always run in triplicate for each standard concentration. In the agglutination assay, failure to obtain the expected result for the last positive PHA-P dilution well constitutes the basis for repeating the analysis. For ELISA, values for standards on a plate must fall within the expected range, based on the means and standard deviation of at least 10 independent assays used to establish a QC range, which is a standard recommendation and best practice.

### 4.4. Assessing PHA Using the Hemagglutination Assay

The hemagglutination assay is considered the most commonly used method for assessing PHA. Main reasons for this include (1) the low level of technical expertise required, (2) low cost, (3) high throughput capacity, and (4) length of time required to complete an assay. However, other than the limitations mentioned in the Introduction, the assay is relatively insensitive and semiquantitative, since the limit of experimental accuracy for this technique is ±1 dilution, corresponding to an error of 25% as summarized in [13]. In our hands, the type of red blood cells (rabbit or human) and the manner in which they are treated after harvest is highly variable as reported in the literature, and there is no clear consensus on a common source or method for treatment, which is critical for method harmonization. For that reason, we propose fresh defibrinated rabbit red blood cells harvested from New Zealand white rabbits that are shipped on ice and that have an expiration date (cat. DRB030, Hemostat Labs, Dixon, CA, USA). In our experience, red blood cell source, treatment, and age must be standardized. In addition, by including a PHA positive control on every agglutination plate over the period of a year, assay drift was noted even in experienced users. This led us to develop the image analysis approach reported in Section 2.5 and Section 2.6 for determining the last positive/first negative serial dilution. Other problems with the use of hemagglutination as an endpoint are summarized (Supplementary Table S5).

### 4.5. Assessing PHA Using an ELISA Assay

Both thyroglobulin and fetuin have been used as targets for active PHA in ELISA assays [3,13]. On theoretical grounds, thyroglobulin is considered to have a higher affinity and specificity for PHA than fetuin [13]. Nonetheless, both targets were evaluated in the development of the ELISA assays reported in Section 2.7 and Section 3.4. Consistent with previous reports, the lower limit of quantification was 15 ng of PHA, corresponding to approximately 30 ppm sensitivity [13]. One difference between the assays is that the secondary antibody concentration was 1:50,000 vs. 1:20,000 for fetuin and thyroglobulin, respectively, using the reagents detailed in Section 2.7. Antibody concentration can impact chromophore saturation. Saturation of the detection chromophore in an ELISA assay occurs when the concentration of the colored end-product exceeds the dynamic range of the plate reader. This results in an inaccurate, artificially high signal that appears to “flatline”, making it impossible to quantify the actual analyte concentration in the sample accurately.

### 4.6. Application of the Proposed Methodology

The literature indicates that 4–5 soaked raw cooked dark red kidney beans are sufficient to induce food poisoning in 1–3 h [5,6]. In the absence of information on which cultivar was ingested, we obtained DRK bean seed from a commercial retail vendor, determined the average weight of 4 seeds (2.44 g), ground and homogenized 4 seeds (in triplicate), took the 10% *w*/*v* supernatant, and subjected it to the hemagglutination assay for range finding and then for the ELISA assay made one dilution from the original extract. The concentration of active PHA was 223 ± 0.07 mg/g of powder (CV < 1%), and the total amount of PHA contained in 4 DRK seeds is 544 mg. Using the hemagglutination assay, the active PHA content is estimated at 327.9 mg/g. Multiplying this number by 2.44, the estimated weight of 4 DRK seeds, would give 800 mg of active PHA associated with acute food poisoning. To our knowledge, ELISA analysis is the first quantitative estimate of an exposure dose for active PHA, which induces classic symptoms of food poisoning within 1–3 h. For several reasons, it cannot be considered a definitive value, as the cultivar and location grown were not identified in [5] and there would be a difference between 4 and 5 DRK seeds of 20%. However, it is a valuable illustration of our approach and broader effort to harmonize methods and determine exposure limits.

Because clinical evidence is emerging that suggests that 1 to 1.5 cups of cooked beans per day have health-beneficial effects [22,23,24,25,26,27,28], and it has been reported that commercially canned cooked beans contain active PHA [19], the proposed assays were applied to several market classes of cooked beans. As shown in canned beans (Table 3), and if, PHA is markedly reduced in canned beans. If the entire can is consumed, which contains 94 g of dry matter, equivalent to 1.5 cups of cooked beans, then exposure to active PHA is approximately 1000-fold lower than the amount associated with acute food poisoning.

### 4.7. Limitations

A primary focus of this work was to propose protocols that would facilitate the standardization of methods for quantifying active PHA content in beans and bean ingredients, e.g., cooked whole bean flour measurement and standardization of protocols across laboratories. Because other laboratories need to examine and further refine these protocols, we did not attempt to validate these approaches across a wide range of bean ingredients, e.g., fractionated flours, or bean-containing food products. Also, while undertaking this work, we identified variations in the PHA content of raw (unprocessed) Pinto bean seed that appear to extend beyond the market classes and cultivars reported in [29,30]. Systematic screening of bean germplasm across market classes, cultivars, growing locations, and seasons is required to identify low-PHA cultivars with favorable agronomic traits that could add value to this crop in the pulse ingredient market.

## 5. Conclusions

Potential sources of variation for measuring active PHA in raw and processed beans were identified. Extraction of samples using a Bead Ruptor Elite homogenizer is proposed to improve consistency and efficiency of soluble protein extraction, followed by centrifugation of homogenates at 15,000× *g* for 15 min to produce clear supernatant fractions suitable for downstream analyses. To increase objectivity, a digital image analysis algorithm was developed to determine endpoints in the hemagglutination assay, and the assay was used to estimate the amount of PHA in a sample. An ELISA assay was used to quantify PHA concentration, and the data were used to estimate exposure dose and safety. It is concluded that combining the hemagglutination assay for range finding with an ELISA assay is an efficient method for determining the actual concentration of active PHA in samples with unknown PHA activity.

## Figures and Tables

**Table 1:** Total Protein Concentration of the Extract.

Method of Homogenization	Mean Protein Conc in Supernatant (mg/mL) ^1^	Std. Dev.
Vertical vortex, 30 s	16.5	0.8
Multi-tube vortex, 60 min	16.8	0.2
FastPrep-24, 6 m/s, 30 s × 10 cycles, 30 s dwell on ice cold acetone between cycles, 30 stainless steel beads	20.6	0.1
Bead Ruptor Elite, 6 m/s, 1 min × 1 cycle, 10 stainless steel beads	23.1	0.9

^1^ mean protein concentration measured by BCA.

**Table 2:** Concentration of PHA in different market classes of raw seed.

Market Class	DRK	WK	BLK	PNT 1	PNT 2	PNT Heidi
HAU	8192	4096	4096	2048	8	1
PHA mg/g dry weight	223.06 ± 0.07	144.0 ± 0.01	86.82 ± 0.02	93.78 ± 0.02	0.39 ± 3.60 × 10^−5^	0.008 ± 2.76 × 10^−6^

BLK: black; DRK: dark red kidney; HAU: hemagglutination units; PHA: phytohemagglutinin; PNT: pinto; WK: white kidney.

**Table 3:** Concentration of PHA in commercially canned market classes.

Market Class	DRK	WK	BLK	PNT
HAU	16	4	64	8
PHA mg/g dry weight	0.0049 ± 1.53 × 10^−7^	0.0047 ± 5.27 × 10^−6^	0.0038 ± 5.59 × 10^−7^	0.0038 ± 1.23 × 10^−7^

BLK: black; DRK: dark red kidney; HAU: hemagglutination units; PHA: phytohemagglutinin; PNT: pinto; WK: white kidney.

## Data Availability

Original contributions presented in this study are included in the Supplementary Materials. Further inquiries can be directed to the corresponding author.

## Supplementary Materials

The following supporting information can be downloaded at: https://www.mdpi.com/article/10.3390/foods14244247/s1, Figure S1: Raw images of polyacrylamide gel electrophoresis (PAGE); Figure S2: Hemagglutination plate layout option 1; Figure S3: Hemagglutination plate layout option 2; Figure S4: Hemagglutination results from raw DRK at different centrifugation speeds; Figure S5: Hemagglutination results of cooked bean samples; Figure S6: Image analysis of hemagglutination negative controls; Figure S7: Hemagglutination of PHA isoforms; Figure S8: Image analysis profile of a commercially available raw DRK bean seed; Figure S9: Representative ELISA plate; Figure S10: PHA-P ELISA standard curve; Table S1: PHA isoform sources; Table S2: Reference table for PHA-P positive control in the hemagglutination assay; Table S3: Reference for unknown samples in the hemagglutination assay; Table S4: Reference for unknown samples in the hemagglutination assay; Table S5: Hemagglutination assay limitations; Protocol S1: Bean sample preparation and hemagglutination protocol; Protocol S2: PHA-P ELISA protocol.

## Abbreviations

The following abbreviations are used in this manuscript:

BCA	Bicinchoninic acid
DRK	Dark red kidney
ELISA	Enzyme-linked immunosorbent assay
HAU	Hemagglutination unit
PAGE	Polyacrylamide gel electrophoresis
PBS	Phosphate-buffered saline
PBSB	Phosphate-buffered saline with bovine serum albumin
PBST	Phosphate-buffered saline with Tween 20
PHA	Phytohemagglutinin
RBC	Red blood cell
