# Supplementary material for: Toward Standardized Measurement of Active Phytohemagglutinin in Common Bean, Phaseolus vulgaris, L
Source: Foods. 2025 Dec 10;14(24):4247. doi: 10.3390/foods14244247 (PMC12732190; doi:10.3390/foods14244247)
Supplement: Supplementary file 1 [file foods-14-04247-s001.zip › Bean Sample Preparation and Hemagglutination Assay Protocol S1.pdf]

Bean Sample Preparation and Hemagglutination Assay Protocol

Prepare in advance:
Phosphate Buffered Saline (PBS), pH 7.4 – used for preparing standards and samples.
Formulation for Buffer is listed on the last page of this protocol.

Sample Preparation & Extraction
All sample extracts are made 10% (w/v) in PBS, pH 7.4
Note: The same extracts prepared and run in the hemagglutination assay are also used for PAGE and the ELISA assay.

1. If analyzing raw seed, skip to step 2. When analyzing cooked bean, the sample is cooked, cooled to room temperature. Cooked bean seed and leachate are homogenized in a Ninja Smoothie Blender (model SS101, SharkNinja, Needham, MA, USA) using extract mode for 30 sec. Homogenized cooked bean is poured into gallon size Ziploc bags, sealed and stored flat at -70°C until frozen. Remove frozen bags of sample and gently break up while in the bag still with a small dead blow hammer. Load the broken sample chunks on a foil lined sample tray, place into the freeze dryer (Harvest Right, Salt Lake City, UT, USA) and freeze dry under vacuum for 24 hrs. Freeze-dried bean is ground into a fine homogenous powder using a coffee grinder and stored at -20 °C in 15 or 50 mL conical tubes until ready for use.
2. Raw dry seed samples are ground to a fine powder, using a designated coffee grinder that can be disassembled and completely cleaned in between samples. Ground material should be able to pass through a 0.5 mm sieve. When grinding, perform several short pulses (5 sec) to prevent excessive warming of the sample.
3. Transfer the ground powder to a 15-50 mL conical tube and freeze at -20°C until ready to use.
4. Remove the sample from freezer and warm to room temperature prior to weighing.
5. Approximately 150 mg of sample powder is weighed into two tared, 2.0 mL screw top O-ring seal bead tubes (cat. 10832, Biospec Products, Barttlesville, OK, USA) containing ten 2.3 mm yttria stabilized zirconia ceramic beads (cat. 11079123zxy, Biospec Products, Barttlesville, OK, USA) per tube and prepared with PBS, pH 7.4 to a total volume of 1500 µL (10% w/v). One tube is labelled rep "A" and the other rep "B". Two replicates ensure there is enough sample for future analyses and in the event that there is an issue with one replicate the second replicate has been processed the exact same way and results should be comparable to the first replicate.
6. Extracts are homogenized using a Bead Ruptor Elite (BRE) Bead Mill Homogenizer (Revvity-Omni International, Waltham, MA, USA) as follows: all tubes are placed in the tube adapter for the BRE with the tube holder adapter placed on top to keep the tubes in place. Holding the adapter securely place all tubes on ice to chill for 1 min. Remove from ice and place back on the BRE instrument and secure, run at 6 m/sec for 30 sec. Transfer the tube adapter with tubes back to the ice for 1 min and repeat the run again for a second time, 6 m/sec for 30 sec. Resulting in a total homogenization time of 1 min. *Note: a FastPrep-24 instrument (MP Biomedicals, Santa Ana, CA, USA) using the same settings listed above can be used as an alternative to the BRE and will achieve similar results.*
7. Remove sample tubes and spin down at 15,000 x g for 15 min (room temp).
8. Supernatant is then transferred to a pre-labelled, 1.5 or 2.0 mL tube. If not using the extract supernatant immediately, freeze at -20 °C. When ready to use, remove the sample and thaw to room temperature prior to use.

Bean Sample Preparation and Hemagglutination Assay Protocol

Preparing the Phytohemagglutinin (PHA-P) positive control

The specific protein form of purified PHA-P solution serves as a positive control of lectin activity and as a reference to calculate amounts of active lectin in the samples.

1. Weigh out approx. 1.6 mg of salt-free PHA-P (cat. L8754, Millipore-Sigma, St. Louis, MO, USA) into a 5 mL conical tube, add PBS to a total volume of 1 mL. To make a larger volume and to ensure greater accuracy when weighing, it is best practice to weigh out 8 mg of PHA-P in a 20 mL glass scintillation vial, then add 4992 µL of PBS resulting in a total volume of 5 mL with an approximate concentration of 1.6 mg/mL (w/v).
2. Swirl to hydrate and mix well all the lyophilized powder.
3. Place the vial into the incubator (37 °C) for 15 min to help aid the PHA-P powder to go into solution. Remove the vial and swirl to mix, if particles are still visible in the solution, place the vial back in the incubator for an additional 10 min.
4. Cool to room temperature, aliquot into desired volumes and freeze at -20 °C. Suggested aliquoting volume is 110 µL in 0.2 mL tubes. Two aliquots are sufficient for running one hemagglutination assay.
5. Pull one aliquot to measure protein concentration using the bicinchoninic acid (BCA) protein determination assay, which is generally considered to be more sensitive with a wider range of protein detection and is more compatible with detergent containing samples, compared to other methods like Bradford or Lowry.

Note: *Protein concentration of PHA-P is supplied at approx. 60-70% from the manufacture. Protein concentration will be less than the concentration of the mixture by weight. A 1.5–1.6 mg/mL preparation of PHA-P by weight will typically result in a protein concentration of approximately 1 mg/mL as measured by BCA. After reconstitution, the PHA-P solution is stable for several weeks at -20 °C. Do not store PHA-P refrigerated as it will lose potency over time.*

Hemagglutination Assay

A. Choosing an appropriate plate layout

The assay is performed using a standard 96-well clear round-bottom plate. Samples are serially diluted in 1/2 pattern across the rows in PBS. Red blood cells are added at equal concentration to all the wells. Agglutinated cells form an amorphous homogenous turbid solution, whereas non-agglutinated RBCs fall to the bottom forming a condensed small round pellet at the base of the well. As shown in the section below, two suggested plate layouts are offered as options to study different types of samples. For example, cooked or suspected low lectin containing samples can most likely be evaluated using a single row of 12 serial dilution wells in a single plate (option 1). However, raw or high lectin containing samples may need more than 12 serial dilution wells (option 2). Regardless of the plate layout, one row can be used for negative control (PBS & RBC solution only without samples) at the expense of one sample row.

Bean Sample Preparation and Hemagglutination Assay Protocol

Option 1	
A	PHA-P-Positive Control A1-12
B	Sample 1 (12 Wells) plate 1 B1-12
C	Sample 2 (12 Wells) plate 1 C1-12
D	Sample 3 (12 Wells) plate 1 D1-12
E	Sample 4 (12 Wells) plate 1 E1-12
F	Sample 5 (12 Wells) plate 1 F1-12
G	Sample 6 (12 Wells) plate 1 G1-12
H	PBS-Negative Control H1-12

Plate 1

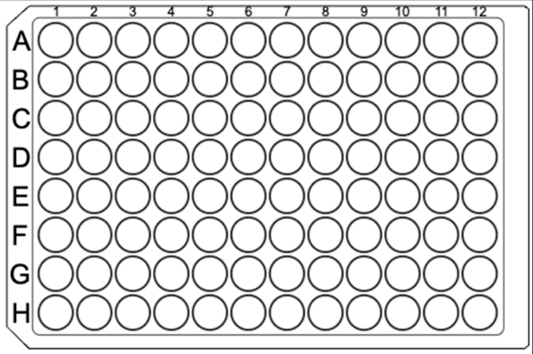

Plate Layout Option 1: Cooked or suspected low lectin containing samples

The PHA-P positive control is in row A and samples are in the remaining rows, B–G with the negative control in row H. A total of 6 samples can be loaded on a single 96-well plate in addition to the PHA-P positive control and negative control (PBS).

Option 2	
A	PHA-P-Positive Control (24 wells) plate 1 A1-12 & plate 2 A1-12
B	Sample 1 (24 Wells) plate 1 B1-12 & plate 2 B1-12
C	Sample 2 (24 Wells) plate 1 C1-12 & plate 2 C1-12
D	Sample 3 (24 Wells) plate 1 D1-12 & plate 2 D1-12
E	Sample 4 (24 Wells) plate 1 E1-12 & plate 2 E1-12
F	Sample 5 (24 Wells) plate 1 F1-12 & plate 2 F1-12
G	Sample 6 (24 Wells) plate 1 G1-12 & plate 2 G1-12
H	PBS-Negative Control (24 wells) plate 1 H1-12 & plate 2 H1-12

Plate 1

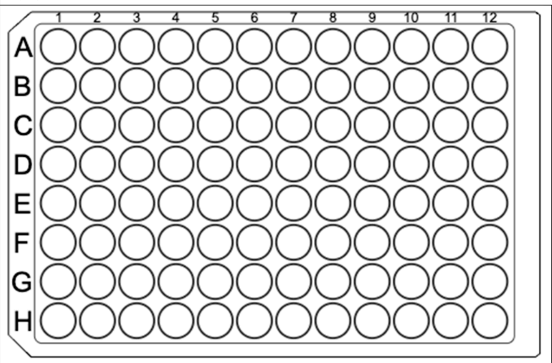

Plate 2

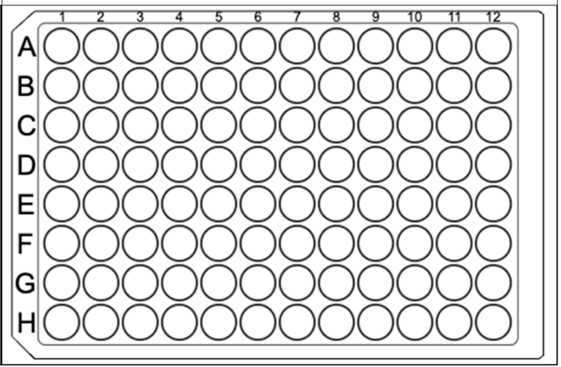

Plate Layout Option 2: Raw or high lectin containing samples

Extend the serial dilutions into a second 96-well plate horizontally, i.e., plate 2 uses the same rows as plate 1, but wells 1–12 in plate 2 become serial dilution wells 13–24. A total of 6 samples can be run in addition to the PHA-P positive control and negative control (PBS).

B. Loading controls, samples and diluent

1. Once the PHA-P standard and samples have been prepared using the methods above, and the plate layout has been selected, label a clear round bottom 96-well plate, designating one row to each sample or standard.
2. Add 12 mL of PBS, pH 7.4, per plate into a reagent reservoir. Using an 8-channel, multichannel pipettor, add 100 µL of PBS using a reverse pipetting technique to all wells according to the intended plate layout, excluding all wells in column 1 of the first plate, i.e., wells designated for the undiluted sample extract.
   Note: For Plate Layout Option 2: exclude column 1 on plate 1 only, as all plate 2 wells (1-12) will have PBS, continuing serial dilutions from plate 1.
3. Load 200 µL of positive control (PHA-P), sample or negative control (PBS) to the first well assigned to each sample/control, using a single channel pipettor, reverse pipetting to ensure accuracy and minimize bubbling due to the viscous nature of the samples.

C. Performing serial dilutions.

Serial dilutions are conducted by aspirating 100 µL from wells in column 1 (half the volume) and mixing with the PBS in wells of column 2 (1:2 dilution) by pipetting the solution up and down 10 times ensuring the solution is adequately mixed in the well. This step is repeated serially, left to right for columns 3–12 (plate layout 1) or columns 3-24 (plate layout 2), thus diluting samples in half from the concentration of the previous (left) well. After mixing the solution in the wells of the last column, 100 µL are discarded, leaving all occupied wells within a row containing 100 µL of diluted solution.

Note: due to the foamy nature of sample extracts, it is recommended to change to fresh pipette tips for each transfer between wells to maintain consistent pipetting accuracy in the assay.

4. a) ***Plate Layout Option 1**:* Perform serial dilutions using a multichannel pipette (forward pipetting technique) by withdrawing 100 µL from wells in column 1 (containing 200 µL of samples and positive control), adding to wells in column 2, mixing 10 times, withdrawing 100 µL, adding to wells in column 3, etc. Repeat this process until reaching the 12th well/column. Mix the sample in the wells of column 12, withdraw 100 µL and discard. A single row of 12 wells in one plate should be sufficient for evaluating suspected low lectin or cooked samples.
   b) ***Plate Layout Option 2**:* Perform serial dilutions as listed for cooked samples (step 4.a. plate layout option 1), but when reaching the 12th well, mix 10 times, withdraw 100 µL and continue serial dilutions in the column 1 of plate 2 and continue mixing and performing serial dilutions until reaching the 12th well of plate 2 (24th well for the sample), withdraw 100 µL and discard.

Note: When finished loading, all occupied wells of the plate should contain a final 100 µL volume of PHA-P or sample dilutions regardless of the plate layout used.

D. Rabbit red blood cells

Using defibrinated rabbit red blood cells (RBCs, cat. DRB030, Hemostat Labs, Dixon, CA, USA), prepare the 2.5% RBC working solution immediately before adding it to the plate to avoid sedimentation of the cells.

5. Make sure the stock bottle of RBCs is tightly closed. Gently invert the bottle several times to mix the solution. Do NOT vortex the bottle as the RBCs are fragile and may lyse.
6. To make a freshly prepared working solution of 2.5% rabbit RBCs in PBS, use a forward pipetting technique and add 300 µL of rabbit RBC stock solution to 11,700 µL of PBS, pH 7.4, in a 15 mL conical tube for a total volume of 12 mL, which is enough volume for all 96 wells in one plate. If running multiple plates, e.g. plate layout 2, multiply by the number of plates being run and scale up accordingly. Use forward pipetting technique. When dispensing the RBCs into the PBS, gently pipet up and down 10–15 times to rinse as much blood as possible from the inside of the pipet tip. Close the conical tube tightly and gently invert the tube 10 times. Do NOT vortex the tube.
7. Gently transfer the solution into a reagent reservoir. Using a multichannel pipette, add 100 µL of the 2.5% rabbit RBC solution to each occupied sample well, using a reverse pipetting technique, starting with the column on the far right of the plate (highest dilution) and working backwards from right to left until reaching the first column of wells. Do NOT touch the contents of the wells with the pipette tip: place the pipette tips over the edge of each well in a column, dispense and wipe any residual on the tips against the right top edge of the wells, allowing it to fall into the respective wells. The resulting volume in all occupied wells should be 200 µL: 100 µL of sample in PBS and 100 µL of RBC solution (1.25% per well).

8. Place the 96-well plate(s) on the plate shaker, up to four at a time (depending on the instrument), and mix at a low speed, e.g., speed 3 out of 10 for 30 sec.
9. Remove the plates from the plate shaker, place flat on the bench and inspect the wells. If any air bubbles are present in the wells, pop them using a 25 G needle. Clean the needle between the wells using a dry Kimwipe.
10. Cover the plate with a 96-well plate lid, with condensation rings, to prevent evaporation.
11. Allow the plates to sit undisturbed at room temperature for a minimum of 2 hrs. prior to photographing. Note: It is highly recommended to photograph the plate again after sitting overnight, which enhances clarity making calls much easier, i.e., last positive well vs first negative well. Overnight photographs are highly recommended when performing image analysis.

E. Photographing and examining the results.

12. Photograph the plate(s) using a backlit lightbox with a white diffusing panel, e.g. Porta-Trace lightbox (Gagne, Inc., Johnson City, NY). A smartphone camera, e.g. iPhone 16 (Apple, Cupertino, CA, USA) is sufficient for taking photographs of the plate(s), but it should be placed directly over the plate on the lightbox with the plate cover off. The smartphone camera should be mounted on a tripod and the camera height raised to 75 cm above the surface of the lightbox to minimize parallax. Both grid and level options should be enabled in the camera app to aid in proper alignment and leveling of the camera prior to image capture. Images are focused by gently tapping on the smartphone screen over the central portion of the plate ensuring that that the photograph will capture a clear view of all wells in the plate. Ambient lighting should be turned off, so that the only source of illumination is from the lightbox, which will eliminate glare. It is highly recommended to use a Bluetooth remote to capture the image as this avoids touching the screen that may cause the image to lose focus. Images are rotated, cropped, and exposure should be increased within the camera app, e.g. 30 points (will vary according to type of device used) prior to importing images to a desktop computer. Hemagglutination plates that have incubated overnight are typically clearer than those incubated for only 2 hrs., thus making the subjective process of delineating between the last positive and first negative wells by eye easier.

Examples:

Below is an example of the dilutions listed for each well for the first row of PHA-P control or sample in plate layout 1 **(Table 1)**. Serial dilutions are listed as 1/X, e.g., 1/2, 1/4, etc. Hemagglutination is reported in hemagglutination units (HAUs), which is the reciprocal of the serial dilution. Report the HAUs of highest dilution, which still causes hemagglutination to occur, i.e. the size and shape of packed RBC dot at the bottom of the well is not the same as those appearing in the negative control wells. If necessary, dilutions can be extended beyond 12 wells as shown in **Table 2**, listing wells 13–24 (see plate layout option 2 above).

Dilutions and hemagglutination units in plate layout option 1.

Plate Well	1	2	3	4	5	6	7	8	9	10	11	12
Serial Dilution	1/1	1/2	1/4	1/8	1/16	1/32	1/64	1/128	1/256	1/512	1/1024	1/2048
HAU	1	2	4	8	16	32	64	128	256	512	1024	2048

Continued serial dilutions and hemagglutination units in plate 2 (plate layout option 2).

Plate Well	13	14	15	16	17	18	19	20	21	22	23	24
Serial Dilution	1/4096	1/8192	1/16384	1/32768	1/65536	1/131072	1/262144	1/524288	1/1048576	1/2097152	1/4194304	1/8388608
HAU	4096	8192	16384	32768	65536	131072	262144	524288	1048576	2097152	4194304	8388608

In the plate row example below, the prepared PHA-P control had a protein concentration of 1.04 mg/mL by BCA, i.e., well A1 contains 104 µg of PHA-P. Serial dilutions of PHA-P are shown plate row A with 2.5% rabbit RBCs in PBS (Figure S3), wells 1–12 (left to right). After 2 hrs., positive hemagglutination can easily be seen in wells A1–9 from, left to right (a sheet of RBCs that covers the entire well). The 10th well shows a smaller pellet of RBCs, but is noticeably larger than the crisp uniform RBC pellets in negative wells 11 & 12 (characteristic small round red dot), making the 10th well also positive for hemagglutination (transitional well). These transitional well RBC dots can appear larger in size than true negative wells and may be round, oblong or have a donut hole in the middle of the RBC pellet. In addition, the well area in transitional wells extending out from the formed pellet may be slightly hazy, i.e. not crisp and clear.

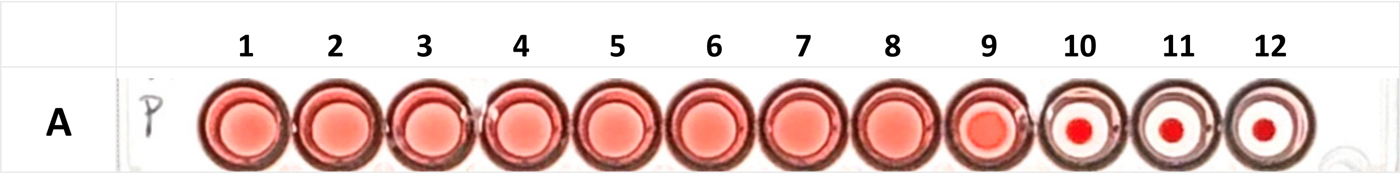

Example of the first row (A) of 96-well plate testing positive control PHA-P in the hemagglutination assay. A total of 100 µL of PBS, pH 7.4 was added to wells 2-12. A 200 µL volume of 1.04 mg/mL concentration of purified PHA-P was added to well 1 (on the left) and serially diluted by removing 100 µL from well 1, transferring to well 2 and mixing 10 times before transferring to well 3, the process repeated until reaching well 12 where 100 µL was withdrawn and discarded. A total 100 µL of 2.5% defibrated rabbit RBC solution in PBS was added to each well for a total volume of 200 µL in each well. The resulting 200 µL in the first well contains 1.25% of RBC fully agglutinated by 104 µg of active PHA-P. Full agglutination is clearly visible in first 9 wells of serial dilutions of PHA-P in PBS using dilution factor 2. Well 10 shows partial agglutination and thus still is considered a positive well. Well 11 shows no signs of agglutination, similar to well 12. Therefore, well 10 is used to report HAUs: since the dilution in well 10 is 1/512, then 1.04 mg/mL of PHA-P shows activity of 512 HAUs. This corresponds to ability of 203.13 ng of PHA-P still able to agglutinate cells in 1.25% RBC solution in 200 µL volume.

F. Image analysis.

Due to the subjective nature of visually distinguishing positive from negative hemagglutination results using the human eye, our laboratory uses image analysis to provide a more objective approach. Captured images from hemagglutination plates incubated overnight are analyzed using an image analysis macro developed by our laboratory.

13. Import color plate images into the open-source ImageJ (ver. 1.5.4) image analysis software (https://imagej.net). The scale is set by measuring the distance from edge to edge of one well in the image using the line tool and the known diameter of the of the bottom of the well according to the manufacturer. Measurements for area and centroid (X, Y location) are also set. Rotate the image slightly to better align rows of wells and the image cropped to the outer edges of the plate prior to converting to 8-bit grayscale.

14. A region of interest (ROI) overlay is drawn on image using the oval tool that covers slightly less than the total inner area of one well starting in the upper left corner of the plate. The initial ROI is duplicated eleven times using the ROI manager, moving each new ROI to the adjacent well to the right, creating a total of 12 ROIs directly superimposed over the 12 wells in the first row of the plate (A). The 12 individual ROIs are combined to create a single ROI, which can be moved from one row to the next. A loop begins where the user is prompted to analyze a row of wells. If yes, the macro continues by creating a binary mask of the combined ROI, which is used to extract the 12 well bottom ROIs from the first row of 12 wells in the plate into a new image with a white background. The threshold range is set at 0-235.
15. Analyze Particles is used to measure the area and centroid of each well bottom. Values are written to the results table in ImageJ and sorted by centroid, with the smallest X values (horizontal) at the top and largest at the bottom, which aligns with the left-to-right position of the 12 wells in a plate row. The results table data is copied to the clipboard and the user is prompted to paste the data into a Microsoft Excel file. The user is then prompted to analyze a row of wells. If yes, the macro repeats the loop allowing the user to move the combined ROI to the next row of wells prior to analysis, and if no, the macro halted execution.
16. Data is graphed in Microsoft Excel with well number on the X axis vs area (mm^2) on the Y axis. Negative controls (PBS and RBCs) must be included along with a PHA-P positive control and samples when performing image analysis. The negative controls are crucial in establishing the mean reference line on the graph where samples transition from positive to negative. We defined a positive hemagglutination area value as three standard deviations above the mean negative area. Edge effect present in the first few serial dilutions of some bean samples resulted in small polygonal hemagglutinated well areas, yielding lower square millimeter area measurements compared to higher serial dilutions, which showed a larger area of hemagglutination before ultimately decreasing to a true negative well. This edge effect is an artifact of the hemagglutination assay and therefore should be ignored when looking area measurements on the graph to determine the last positive well vs first negative well (Figure S6b). The ImageJ macro for image analysis of hemagglutination plates is freely available for use (https://github.com/mcginleyj/hemagglutination).

Equipment, Supplies & Reagents

Reagents needed:

- Salt-free Phytohemagglutinin (PHA-P) lectin from Phaseolus vulgaris-red kidney bean (cat. L8754, Milipore-Sigma, St. Louis, MO, USA)
- Rabbit defibrinated whole blood (cat. DRB030, Hemostat Laboratories, Dixon, CA, USA)
- Sodium chloride
- Potassium chloride
- Sodium phosphate dibasic
- Potassium phosphate monobasic
- Adjust PBS to pH 7.4, if needed

Equipment & Supplies needed:

- Adjustable Electric Herb, Spice, Coffee Bean, Espresso Grinder with 2 Removable Stainless Steel, Shardor. (Amazon https://a.co/d/eNatbY9)
- Ninja Smoothie Blender (Shark-Ninja, Amazon https://a.co/d/7K2wKQh)
- Gallon size Ziploc freezer bags
- Small dead blow hammer (Amazon https://a.co/d/iUYkMly)

- Freeze Dryer (Harvest Right, Salt Lake City, UT, USA)
- Bead Ruptor Elite, Bead Mill Homogenizer (Cat. 19-042E, Revvity-Omni International, Waltham, MA, USA)

Equipment & Supplies needed (continued):
- 2.0 ml screw cap with o-ring seal, microcentrifuge vials (cat. 10832, Biospec Products, Bartlesville, OK, USA)
- 2.3 mm ceramic beads-yttria stabilized, zirconia beads (cat. 11079123zxy, Biospec Products, Bartlesville, OK, USA)
- Titer Plate Shaker (4826, Lab-line Instruments, Inc., Melrose Park, IL, USA)
- Porta-trace lightbox (cat. 1012-2L, Gangne, Inc., Johnson City, NY, https://gagneinc.com/product/stainless-steel-led-lightboxes/)
- Bench top vortex
- Plate Reader/Spectrophotometer (SpectraMax M5, Molecular Devices, San Jose, CA, USA)
- pH meter or pH strips
- 20 mL glass scintillation vial
- Incubator, set to 37 °C. Use an independent temperature probe to ensure correct temperature.
- Various microcentrifuge tubes, 0.5 - 2.0 mL
- Various sized conical tubes, 5, 15 & 50 mL
- Gloves, lab coat, eye protection
- 25G 5/8 inch needle (cat.14826AA, Fisher Scientific, Waltham, MA, USA)
- Kimwipes
- Clean absorbent paper towels (cat. 0666632B, Fisher Scientific, Waltham, MA, USA)
- Pipettors capable of 10, 20, 200, 1000, 5000 µL (Gilson P10, P20, P200, P1000 & Eppendorf 5000 µL)
- Multi-channel pipettors capable of 50 & 100 µL (Rainin LTS 8-channel 50 µL, Eppendorf 8-channel 100 µL)
- Pipet tips compatible with types and sizes above
- Clean glass bottles for buffer solutions
- Stir plate and stir bars
- Volumetric flask 1L or 2L
- A four-point balance (capable of accurate weight to the mg)
- Weigh boats and spatulas
- Ultrapure water (Milli-Q water purification system, Millipore-Sigma, Inc., St. Louis, MO, USA)
- Clear Round Bottom 96 well plates (cat. 12565214, Fisher Scientific, Waltham, MA, USA)
- Reagent Reservoirs 50 mL (cat. 89094-674, Avantor-VWR, Radnor, PA, USA)
- open-source ImageJ (ver. 1.5.4) image analysis software (https://imagej.net)
- ImageJ macro (https://github.com/mcginleyj/hemagglutination)

Buffer Formulation and Preparation:

Phosphate Buffered Saline (PBS), pH 7.4 Preparation:

For 1 liter of 1X PBS, prepare as follows:

Dissolve the following in 800 ml of Milli-Q H_2O:
8g NaCl (sodium chloride)
0.2g KCl (potassium chloride)
1.44 g Na_2HPO_4 (Sodium phosphate dibasic)
0.24 g KH_2PO_4 (potassium phosphate monobasic)
Check the pH with a pH meter, if necessary, pH can be adjusted to pH 7.4.
(use hydrochloric acid (HCl) to lower pH or sodium hydroxide (NaOH) to increase pH).
Adjust to final volume in a volumetric flask.

Bean Sample Preparation and Hemagglutination Assay Protocol

Store at room temperature.
*If you need sterile PBS, sterilize by autoclaving (20 min at 121°C, liquid cycle)
To make Phosphate Buffered Saline pH 7.4 with 0.05% Tween 20 (PBST) -add 1ml of Tween 20 to 2L of PBS.
