# Supplementary material for: Toward Standardized Measurement of Active Phytohemagglutinin in Common Bean, Phaseolus vulgaris, L
Source: Foods. 2025 Dec 10;14(24):4247. doi: 10.3390/foods14244247 (PMC12732190; doi:10.3390/foods14244247)
Supplement: Supplementary file 1 [file foods-14-04247-s001.zip › PHA-P ELISA Protocol S2.pdf]

PHA-P ELISA Protocol

PROTOCOL

For a detailed list of equipment, supplies & reagents needed, see the end of this protocol.

Unless specified otherwise, reagents are poured into a reagent reservoir and a multichannel pipettor is used throughout to apply reagents using a reverse pipetting technique, 100 µL in each well, adhesive film for micro plates is applied to the top of the plate after loading each reagent, plates are shaken on a titer plate shaker, speed 3 for 30 sec, and incubations are done in 37 °C incubator for 1 hr.

Preparation:

Prepare in advance:

- Phosphate Buffered Saline (PBS), pH 7.4 – used for preparing standards and samples
- Phosphate Buffered Saline, pH 7.4 with 0.05% Tween 20 (PBST) – used for all plate washing steps.
- 50 mM (0.05 M) Carbonate-Bicarbonate Buffer pH 9.8 (CBB) – used for the plate coating process.

Prepare early the day of running the assay:

- Phosphate Buffered Saline pH 7.4 with 0.5% BSA (PBSB) - for blocking, a minimum of 13.5 mL/plate is needed.
- Phosphate Buffered Saline pH 7.4 with 0.25% BSA-(PBSB) for preparation of primary and secondary antibodies, a minimum of 27 mL/plate is needed.
  Note: Use a bovine serum albumin (BSA) that is protease-free and globulin-free. When the BSA is mixed with PBS, the resulting PBSB solution will be very foamy and will need time for the bubbles to settle prior to use.

Prepare Plate Washer:

1. Empty the plate washer waste container.
2. Fill the plate washer buffer bottles and Milli-Q water bottle(s).
3. Turn on plate washer allow it to initialize. Run new buffer prime for bottles that have been filled. If no bottles were filled with new buffer, then execute a prime run to ensure fluids are flowing properly through the tubing and manifold.
4. Lastly, run a wash protocol x 3 to further prime the instrument.

Coating the plate

The plate can be coated either the afternoon before or on the day of running the assay:

5. Coat the Nunc Maxisorp ELISA plate with 8 µg/ml of either fetuin or porcine thyroglobulin in 50 mM carbonate-bicarbonate buffer, pH 9.8, 100 µL in each well. Seal the plate using adhesive plate seal, shake the plate briefly and either place in Ziploc bag and incubate overnight at 4 °C or incubate the sealed plate at room temp for a minimum of 1 hour on the bench top.
6. Check the incubator temperature using an independent K-type thermocouple probe. Temp should be 37 °C.
7. If the plate was coated overnight at 4 °C, remove the plate from the refrigerator/cooler and allow the sealed plate to warm up to room temp for a minimum of 1 hr. If the plate was coated the morning of the assay, then move on to the next step after the 1 hr. incubation at room temp.

PHA-P ELISA Protocol

Preparation of standards and samples:
While the plate coating is incubating for 1 hr., prepare standards and samples using a minimum volume of 500 µL.

8. **Standard curve:** use phytohemagglutinin (PHA-P) reconstituted to approximately 1 mg/mL in PBS, protein concentration confirmed previously by BCA. Dilute the 1 mg/mL stock PHA-P to the following concentrations: 0, 12.5, 25. 50, 100, 200, 300 ng/100 µL in PBS. *Note:* the zero concentration will be straight PBS only, no PHA-P, and will serve as a blank.
9. **Samples:** each sample is diluted in PBS using the results from the hemagglutination assay as a guide for preparing two target dilutions per sample. We have found that for low lectin containing samples, a 1/1 or 1/2 dilution works best, and for samples containing a high level of lectin hemagglutination activity, the first and second negative wells are used as target dilutions, e.g. well 13 (1/4096) and well 14 (1/8192), to ensure the sample concentration will fall within the range of the standard curve. *Note: any volume over 2 mL is pipetted with a P1000 and any volume under 2 mL is pipetted with a P200 or P20, which maintains greater accuracy. For more detailed information on preparation of standards and samples see pages 5-6, Prepare Standards and Samples section.*

Before washing the ELISA plate make sure the plate washer has been turned on and primed!

10. **Wash X 3** using PBST.
11. Invert the plate on clean paper towel to remove any excess buffer.

Blocking

12. Block with 0.5% bovine serum albumin (protease-free, globulin-free BSA) in PBS (PBSB), a minimum volume of 13.5 mL/plate will be needed. Pipette 100 µL in each well, seal the plate using a new adhesive plate seal, shake plate briefly, incubate for 1 hr. at 37 °C. If protease-free, globulin-free BSA is not available, 5% normal mouse serum in PBS can be used as a blocking alternative.
13. Using the plate washer, run the **Aspirate** program to remove the block, **<u>DO NOT WASH THE PLATE</u>!**
14. Invert the plate on clean paper towel to remove any excess buffer.

Loading standards and samples
While the plate is in BSA block, continue preparing sample dilutions.

15. Once the standards and samples have been prepared using the appropriately chosen dilutions, load a minimum of 140 µL in each well, in triplicate into a new, clear, round bottom intermediate plate using a single channel pipettor. The use of an intermediate plate will greatly reduce the amount of time necessary to load standards and samples into the ELISA plate, thus protecting the ELISA plate from drying out. *Note: While flat bottom plates can be used, round bottom plates, the same type used for hemagglutination, work best as an intermediate plate for aspirating and transferring samples to the ELISA plate.*
16. Transfer the standards & samples from the intermediate plate to the ELISA plate using a multichannel pipet. Load standards and diluted samples, **100 µL in each well in triplicate**, seal the plate using a new adhesive plate seal, shake the plate briefly (speed 3 for 30 sec) and incubate for 1 hr. at 37 °C.

Prepare antibodies:
While the standards and samples are incubating on the plate, prepare the antibody dilutions.

17. Dilute the **primary antibody**, Rabbit-Anti-PHA-P (E+L) Final concentration: 4 µg/mL in 0.25% PBSB.

18. Dilute the **secondary antibody**, Mouse anti-rabbit IgG conjugated to horseradish peroxidase (HRP). If the plate was coated with fetuin, dilute the secondary antibody to 1:50,000 in 0.25% PBSB. If the plate was coated with thyroglobulin, dilute the secondary antibody to 1:40,000 in 0.25% PBSB.
    Note: due to the high dilution factor of the secondary antibody use an intermediate dilution, e.g. 1:500 to make the final dilution.

19. Pull the Stable Peroxide Buffer from the refrigerator and warm to room temp.

20. **Wash X 4** with PBST.
21. Invert the plate on clean paper towel to remove any excess buffer.

Primary antibody

22. Add primary antibody, rabbit-anti-PHA-P (E+L), 100 µL in each well, seal the plate using a new adhesive plate seal, shake plate briefly, and incubate for 1 hr. at 37 °C.

23. **Wash X 4** with PBST.
24. Invert the plate on clean paper towel to remove any excess buffer.

Secondary antibody

25. Add secondary antibody, Mouse anti-rabbit IgG conjugated to horseradish peroxidase (HRP). Add 100 µL in each well, seal plate using a new adhesive plate seal, shake plate briefly, and incubate for 1 hr. at 37 °C.

Substrate

26. Once the incubation of the secondary antibody is almost done pull OPD substrate from freezer and weigh out into a 15 mL conical tube, approximately 6 mg and cover the tube with foil (OPD is light sensitive).

    OPD substrate, prepare immediately before use:

27. Dissolve 6 mg of OPD in 10.8 mL of milli-Q water. Add 1.2 mL of Stable Peroxide Buffer to the dissolved OPD solution. (Alternatively, dissolve OPD to 0.5-1.0 mg/mL in a buffer containing 0.05 M citric acid, 0.05 M sodium phosphate; pH 5 and then add 1 µL of 30% hydrogen peroxide per 1 mL of substrate).
28. Remove plate from incubator and **Wash X 6** with PBST.
29. Invert the plate on clean paper towel to remove any excess buffer.
30. Add 100 µL of the OPD solution to each microplate well, seal plate using a new adhesive plate seal, shake plate briefly. Once shaken, place the plate in the plate reader and close to protect from light and **incubate at room temperature for 30 min**.
31. To stop the reaction, remove the plate seal and add 50 µL of 2.5M sulfuric acid (5 Normal) to each well using a multichannel pipette with filter tips.
32. Read the plate using endpoint determination. Plate reader settings: wavelength 490 nm, path check OFF and Shake ON (5 sec prior to read).
    Note: For non-stopped assays, measure the absorbance at 450 nm.
33. Copy and paste the read data values to a text file or Excel file.

34. Use GraphPad Prism or other appropriate software to generate a standard curve. Calculate CVs of the absorbance values in triplicate. Ideally, CVs < 5 is ideal, but anything < 20 is acceptable. Perform baseline correction by subtracting the absorbance values of the PBS blank from all standards and samples. Perform linear regression using the baseline corrected data to plot absorbance values of the PHA-P standards vs the concentration in ng/µL. The R square value should be > 0.99. If not, remove one or two of the standards to improve linearity. Once the standards have been analyzed, add the sample absorbance values and run linear regression again to interpolate sample concentration based on absorbance values as related to the standard curve.
Note: ideally, sample absorbance values should be close to 1.0. If absorbance values are < 0.046 (too dilute) or > 1.4 (too concentrated) then repeat the ELISA using more concentrated or more dilute samples. Absorbance values > 2.0 are too concentrated and must be repeated using a higher dilution.

Equipment, Supplies & Reagents needed
Buffers
Prepare in advance**:**
- Phosphate Buffered Saline pH 7.4, (PBS)
- Phosphate Buffered Saline Tween pH 7.4, 0.05% Tween 20 (PBST)
- 50 mM (0.05M) Carbonate-Bicarbonate Buffer pH 9.8 (CBB)

Prepare the day of:
- Phosphate Buffered Saline pH 7.4, with 0.5% BSA (PBSB) – used for blocking, a minimum of 13.5 mL/plate is needed.
- Phosphate Buffered Saline pH 7.4, with 0.25% BSA (PBSB) – used for antibody dilutions, a minimum of 27 mL/plate is needed.

Reagents needed:
- Bovine serum albumin, protease-free, globulin-free BSA (Sigma A7030)
- Sodium bicarbonate
- Sodium carbonate (anhydrous)
- Sodium chloride
- Potassium chloride
- Sodium phosphate dibasic
- Potassium phosphate monobasic
- Tween 20
- Adjust pH of PBS to 7.4

Plate coating
- Fetuin (cat. F3004, Millipore-Sigma, St. Louis, MO, USA), solutions are stable for ~2 weeks at 2-8 °C, and several months when aliquoted and stored at -20 °C.
  OR
- Porcine Thyroglobulin (cat. T1126, Millipore-Sigma, St. Louis, MO, USA).

Note: We have found Fetuin to be more sensitive than thyroglobulin.

PHA-P ELISA Protocol

Standards & Samples

For each sample or standard prepare a minimum of 500 µL in order to load a minimum volume of 140 µL in triplicate into the intermediate plate, both standards and samples are diluted in PBS only, no BSA or Tween 20.

Standards

- Phytohemagglutinin (PHA-P, salt-free, cat. L8754 Millipore-Sigma, St. Louis, MO, USA), lectin from Phaseolus vulgaris-red kidney bean.

Weigh out approx. 1.6 mg of PHA-P into a 5 mL conical tube, add PBS to a total volume of 1 mL. To make a larger volume, ensuring greater accuracy when weighing, it is preferable to weigh out 8 mg of PHA-P in a 20 mL glass scintillation vial, then add 4992 µL of PBS resulting in a total volume of 5 mL with an approximate concentration of 1.6 mg/mL. Vortex for 30 sec, place into the incubator (37 °C) for 15 min (to ensure the PHA-P is in solution), remove and vortex 30 sec (if particles are visible in the solution, place the vial back in the incubator for an additional 10 min). Cool to room temperature and aliquot into desired volumes and freeze at -20 °C. Keep one aliquot to measure protein concentration using the bicinchoninic acid protein determination (BCA) assay, which is more accurate than the Bradford assay.
Note: the purity of this PHA-P is usually 60-70%, so a weight of 1.6 mg/mL will likely result in protein concentration of approximately 1 mg/ml as measured by BCA.
For the standard curve, dilute the Phytohemagglutinin (PHA-P) stock solution of ~1 mg/mL in PBS. Make standards, by diluting the PHA-P stock concentration from ~1 mg/mL to 0, 12.5, 25. 50, 100, 200, 300 ng/100 µL in PBS.

Samples

- Sample extracts are made 10% w/v in PBS, homogenized using a Bead Ruptor Elite (BRE).

The same extracts prepared for and run in the hemagglutination assay are also used for the ELISA assay. Briefly, samples are ground to a fine powder, 150 mg of sample powder is weighed in into 2.0 ml screw top bead tubes containing ten 2.3 mm ceramic beads per tube and prepared with PBS pH 7.4 to a total volume of 1500 µL (10% w/v). Extracts are then homogenized using the BRE as follows, all tubes are placed in the tube adapter and first chilled for 1 min on ice, then run at 6 m/sec for 30 sec, removed from the BRE, placed on ice for 1 min, and run again for a second time, 6 m/sec for 30 sec. Samples are then centrifuged at 15,000 x g for 15 min (room temp) and supernatant is transferred to a 1.5 or 2.0 mL tube. Use the results from the hemagglutination assay as a guide for preparing two target dilutions per sample. We have found for low lectin containing samples, diluting 1:1 or 1:2 dilution works best. For samples containing a high level of lectin hemagglutination activity, the first and second negative wells are used as target dilutions, e.g. well 13 (1/4096) and well 14 (1/8192) in PBS, to ensure the sample concentration will fall within the range of the standard curve. If the result(s) still fall outside the standard curve then repeat using a higher dilution of the sample.

Antibodies

Primary Antibody:

- Rabbit-Anti-PHA-P (E+L), (cat. AS-2300-1, Vector Laboratories, Newark, CA, USA). Supplied as liquid and exp ~1 yr see vial for exp date. Store at 2-8 °C. Dilution: 4 µg/mL in 0.25% BSA (PBSB).

Secondary Antibody:

- Mouse anti-rabbit IgG conjugated to horseradish peroxidase (cat. 211-035-109, Jackson Immuno Research, West Grove, PA, USA). Good for 6-8 weeks when reconstituted and stored at 4 °C. Good for

1 yr when reconstituted, aliquoted and frozen at -70 °C. *Note:* Dilution of the secondary changes according to coating of the plate (use a 1:500 intermediate dilution to make the final dilution):
Fetuin - dilute the secondary to a final concentration of 1:50,000
Thyroglobulin - dilute to a final concentration of 1:20,000.

Substrate For Colorimetric Reaction

- O-phenylenediamine Dihydrochloride substrate (OPD, cat. 34005, Thermo Fisher Scientific, Waltham, MA, USA)
- Stable Peroxide Buffer (cat. 34062, Thermo Fisher Scientific, Waltham, MA, USA)

Equipment & Supplies

- Plate Washer (ELX50, BioTek Instruments, Winooski, VT, USA)
- Plate Reader/Spectrophotometer (SpectraMax M5, Molecular Devices, San Jose, CA, USA)
- Titer Plate Shaker (4826, Labline Instruments, Melrose Park, IL, USA)
- Bench top vortex
- pH Meter or pH strips
- 20 mL glass scintillation vial
- Incubator, set to 37 °C. Use an independent temperature probe to ensure correct temperature.
- Various microcentrifuge tubes, 0.5 - 2.0 mL
- Various conical tubes 5, 15 & 50 mL
- Gloves, Lab coat, Eye protection
- Clean absorbent paper towels (cat. 0666632B, Fisher Scientific, Waltham, MA, USA)
- Pipettors capable of 10, 20, 200, 1000, 5000 µL (Gilson P10, P20, P200, P1000 & Eppendorf 5000 µL)
- Multi-channel pipettors capable of 50 & 100 µL (Rainin LTS 8-channel 50 µL, Eppendorf 8-channel 100 µL)
- Pipet tips compatible with types and sizes above.
- Clean bottles for buffer solutions
- Stir plate, stir bar
- Volumetric flask 1L or 2L
- Balance (capable of accurate weight to the mg)
- Weigh boats and Spatulas
- Ultrapure water (Milli-Q water purification system, Millipore-Sigma, St. Louis, MO, USA)
- Foil to wrap around tube to protect OPD from light
- Adhesive Film for Micro plates non-sterile (cat. 60941-062, Avantor-VWR, Radnor, PA, USA)
- Clear Flat-Bottom Immuno Nonsterile 96-Well Plate, MaxiSorp surface treatment, Nunc ELISA plate (cat.442404, Thermo Fisher Scientific, Waltham, MA, USA)
- Clear Round Bottom 96 well plates (cat. 12565214, Fisher Scientific, Waltham, MA, USA)
- Reagent Reservoirs 50 mL (cat. 89094-674, Avantor-VWR, Radnor, PA, USA)

PHA-P ELISA Protocol

Buffer Formulations and Preparation:

50 mM (0.05M) Carbonate-Bicarbonate Buffer (pH 9.2 to 10.6) Preparation:

Commonly used for various immunoassay applications and for many protein and antibody conjugation procedures, including ELISA, which require experimental surface coatings. It has good buffering capacity and is easy to prepare, with excellent shelf life.
To prepare 1 L of Carbonate-Bicarbonate Buffer (pH 9.2 to 10.6):

Required components.

Component	Amount	Concentration
Sodium bicarbonate (mw: 84.01 g/mol)	2.409 g	0.02868 M
Sodium carbonate (anhydrous) (mw: 105.99 g/mol)	2.26 g	0.02132 M

Prepare 800 mL of distilled water in a suitable container.
Add 2.409 g of Sodium bicarbonate to the solution.
Add 2.26 g of Sodium carbonate (anhydrous) to the solution.
Add distilled water until the volume is 1 L.

Phosphate Buffered Saline (PBS), pH 7.4 Preparation:

For 1 liter of 1X PBS, prepare as follows:
Dissolve the following in 800 ml of Milli-Q H_2O:
8g NaCl (sodium chloride)
0.2g KCl (potassium chloride)
1.44 g Na_2HPO_4 (Sodium phosphate dibasic)
0.24 g KH_2PO_4 (potassium phosphate monobasic)

Buffer Formulations and Preparation: (Continued…)

Check the pH with a pH meter, if necessary, pH can be adjusted to pH 7.4.
(use hydrochloric acid (HCl) to lower pH or Sodium hydroxide (NaOH) to increase pH).
Adjust to final volume in a volumetric flask.
Store at room temperature.
*If you need sterile PBS, sterilize by autoclaving (20min, 121°C, liquid cycle)
To make Phosphate Buffered Saline pH 7.4 with 0.05% Tween 20 (PBST) -add 1ml of Tween 20 to 2L of PBS.

2.5M Sulfuric Acid-Stop Reagent

Density: 1.84 g/mL, Formula weight: 98.08 g/mol, Weight percentage: 95.0 % w/w
Desired Final Volume: 1000ml
Desired concentration: 2.5 Molar
Stock solution of Sulfuric Acid is calculated to be 17.822 M based on a density of 1.84 g/mL, a formula weight of 98.08 g/mol, and a concentration of 95% w/w.
To make a 2.5 M solution, **slowly** add 140.275 mL of your stock solution to 250 mL deionized water.
Adjust the final volume of solution to 1000 mL with deionized water.
