# Supplementary material for: Toward Standardized Measurement of Active Phytohemagglutinin in Common Bean, Phaseolus vulgaris, L
Source: Foods. 2025 Dec 10;14(24):4247. doi: 10.3390/foods14244247 (PMC12732190; doi:10.3390/foods14244247)
Supplement: Supplementary file 1 [file foods-14-04247-s001.zip › Supplementary Materials V10.pdf]

Table S1. PHA Isoform Sources

PHA Isoform	Description	Vendor	Cat.
P	Mixture of E & L isoforms 1	Sigma	L8754
E	Erythroagglutinin 1	Sigma	L8629
L	Leucoagglutinin 1	Sigma	L2769
M	Mucoprotein 1	Sigma	L8902
E	Erythroagglutinin 2	Vector	L-1120-5
L	Leucoagglutinin 2	Vector	L-1110-5

[1] All isoforms from Sigma, (Millipore-Sigma, St. Louis, MO, USA) were salt-free lyophilized powders; [2] Isoforms from Vector (Vector Laboratories, Newark, CA, USA) were provided in solution, ready to use.

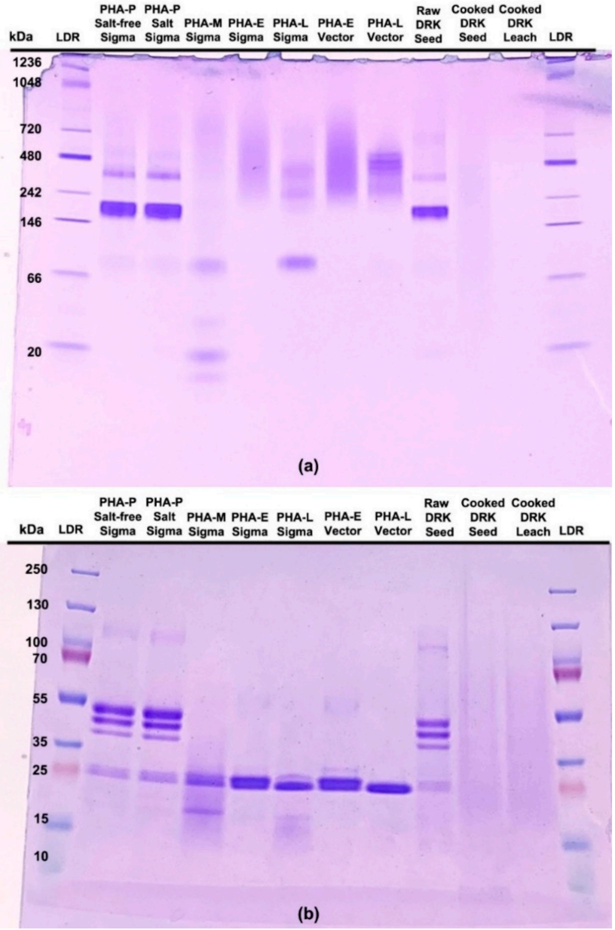

Figure S1. Raw images of polyacrylamide gel electrophoresis (PAGE): (**a**) Native PAGE depicting different isoforms of PHA from two different vendors along with raw and cooked dark red kidney (DRK) bean seed and cooked DRK leachate; (**b**) SDS PAGE depicting the same samples as listed in the native gel, but in the denatured form.

Option 1	
A	PHA-P-Positive Control A1-12
B	Sample 1 (12 Wells) plate 1 B1-12
C	Sample 2 (12 Wells) plate 1 C1-12
D	Sample 3 (12 Wells) plate 1 D1-12
E	Sample 4 (12 Wells) plate 1 E1-12
F	Sample 5 (12 Wells) plate 1 F1-12
G	Sample 6 (12 Wells) plate 1 G1-12
H	PBS-Negative Control H1-12

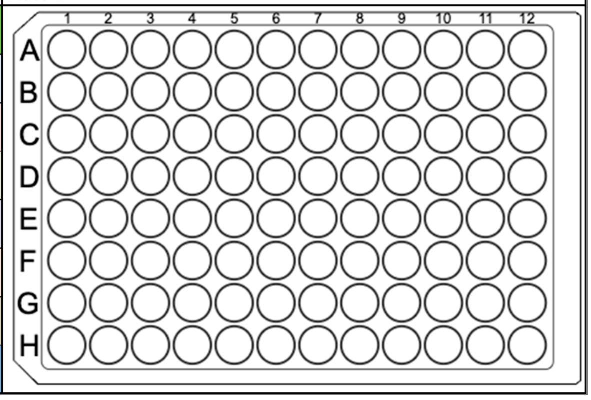

Figure S2. Hemagglutination plate layout option 1: Cooked or suspected low lectin containing samples The PHA-P positive control is in row A and samples are in the remaining rows, B–G with the negative control in row H. A total of 6 samples can be loaded on a single 96-well plate in addition to the PHA-P positive control and negative control (PBS).

Option 2	
A	PHA-P-Positive Control (24 wells) plate 1 A1-12 & plate 2 A1-12
B	Sample 1 (24 Wells) plate 1 B1-12 & plate 2 B1-12
C	Sample 2 (24 Wells) plate 1 C1-12 & plate 2 C1-12
D	Sample 3 (24 Wells) plate 1 D1-12 & plate 2 D1-12
E	Sample 4 (24 Wells) plate 1 E1-12 & plate 2 E1-12
F	Sample 5 (24 Wells) plate 1 F1-12 & plate 2 F1-12
G	Sample 6 (24 Wells) plate 1 G1-12 & plate 2 G1-12
H	PBS-Negative Control (24 wells) plate 1 H1-12 & plate 2 H1-12

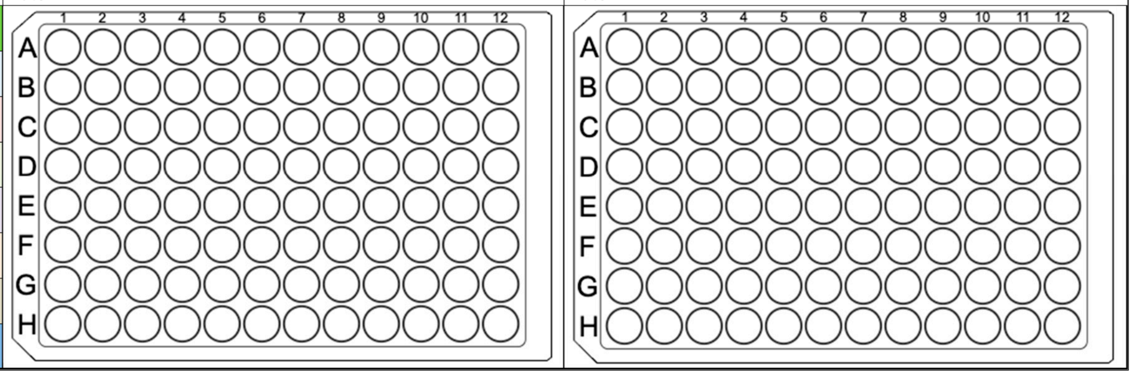

Figure S3. Hemagglutination plate layout option 2: Raw or suspected high lectin containing samples have serial dilutions extended into a second 96-well plate horizontally, i.e., plate 2 uses the same rows as plate 1, but wells 1–12 in plate 2 become serial dilution wells 13–24. A total of 6 samples can be run in addition to the PHA-P positive control and negative control (PBS).

Row	Samples	Last + Well by Eye	HAUs
A	**PHA-P (salt-free) in PBS** BCA Conc. 1.13 mg/ml. Serial dilutions starting at 1:1 A1-24.	10	512
B	**1 - Raw DRK Extract Centrifuged 1000 x g 3 min**. Serial dilutions starting at 1:1 B1-24.	13	4096
C	**3 - Raw DRK Extract Centrifuged 7500 x g 8 min**. Serial dilutions starting at 1:1 C1-24.	13	4096
D	**5 - Raw DRK Extract Centrifuged 15000 x g 15 min**. Serial dilutions starting at 1:1 D1-24.	13	4096
E	**7 - Raw DRK Extract Centrifuged 20000 x g 20 min**. Serial dilutions starting at 1:1 E1-24.	13	4096

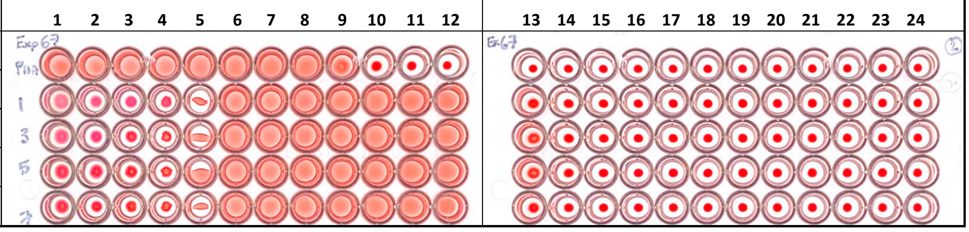

Figure S4. Hemagglutination results from raw DRK at different centrifugation speeds, e.g., 1,000 x g for 3 min, 7,500 x g for 8 min, 15,000 x g for 15 and 20,000 x g for 20 min. Sample extraction processing by vortexing (not bead mill homogenizer), results determined by eye. HAUs are the same and there is not much difference visually except in the first and last well.

Hemagglutination	Plate 1 (12 wells), serial dilutions 1-12
Row	1 2 3 4 5 6 7 8 9 10 11 12
A	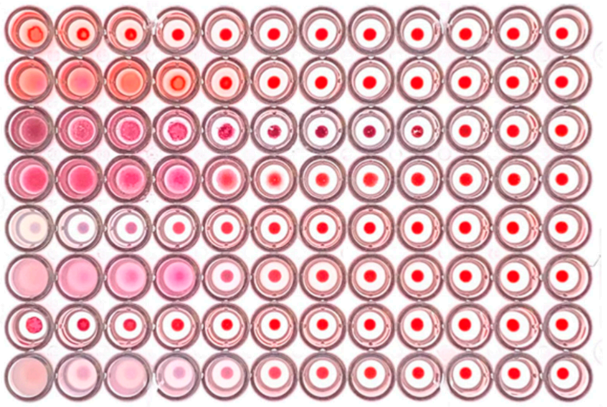
B	
C	
D	
E	
F	
G	
H	

Figure S5. Hemagglutination results of cooked bean samples. Sample extracts were prepared using vortex only and centrifuged at 1000 x g for 3 min. Particulates remaining in the low speed centrifuged extracts resulted in serial dilution wells with higher opacity, which made determination of HAU calls by eye extremely difficult. Extracts prepared in this manner are not suitable for image analysis.

96 well plate exluding peripheral wells											
1	2	3	4	5	6	7	8	9	10	11	12
13	14	15	16	17	18	19	20	21	22	23	24
25	26	27	28	29	30	31	32	33	34	35	36
37	38	39	40	41	42	43	44	45	46	47	48
49	50	51	52	53	54	55	56	57	58	59	60
61	62	63	64	65	66	67	68	69	70	71	72
73	74	75	76	77	78	79	80	81	82	83	84
85	86	87	88	89	90	91	92	93	94	95	96

(**a**)

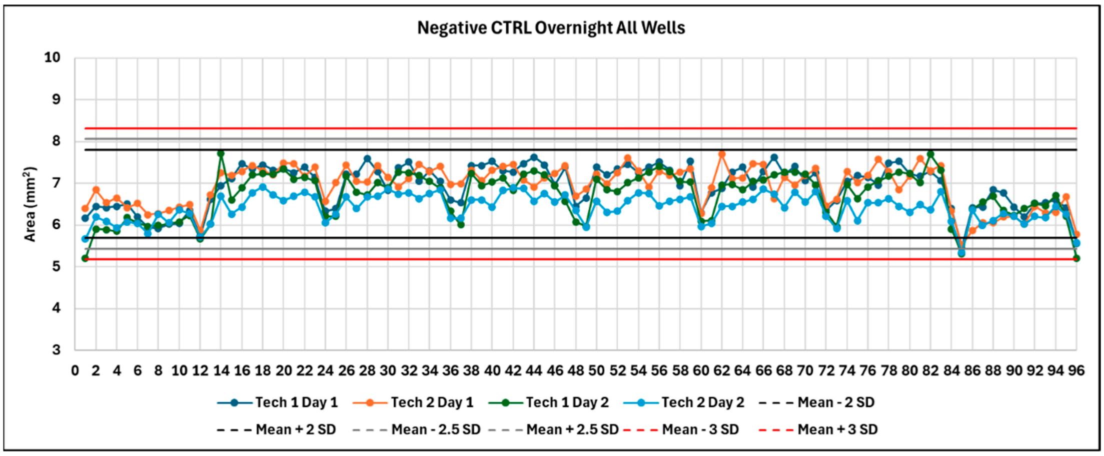

(**b**)

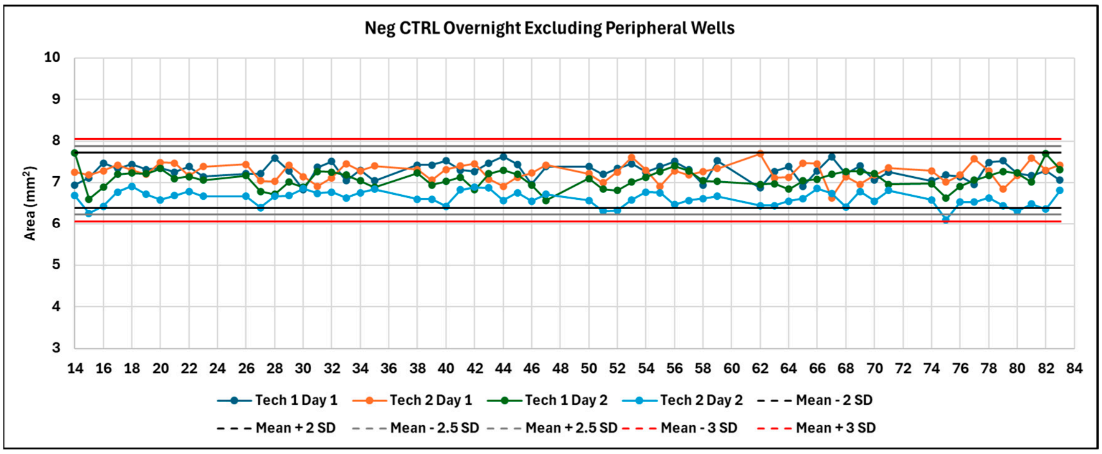

(**c**)

Figure S6. Image analysis of hemagglutination negative controls. (**a**) 96 well hemagglutination plate layout depicting peripheral wells shaded red and interior wells shaded green; (**b**) area measurements of all 96 wells of hemagglutination plates by two techs over two days with SD indicated as the mean ± 2, 2.5 and 3 SD; (**c**) area measurements of hemagglutination plates excluding peripheral wells by two techs over two days with SD indicated as the mean ± 2, 2.5 and 3 SD; Neg CTRL: negative control.

Row	PHA Isoform	Last + well	HAUs	1	2	3	4	5	6	7	8	9	10	11	12
A	**PHA-M (Sigma)** BCA conc. 0.64 mg/ml. Serial dilutions startimg 1:1 A1-24	9	256												
B	**PHA-L (Sigma)** BCA conc. 0.69 mg/ml. Serial dilutions startimg 1:1 B1-24	7	64												
C	**PHA-E (Sigma)** BCA Conc. 0.68 mg/ml. Serial dilutions startimg 1:1 C1-24	11	1024												
D	**PHA-P (Sigma, salt-free)** , BCA conc. 0.69 mg/ml. Serial dilutions startimg 1:1 D1-24	8	128												

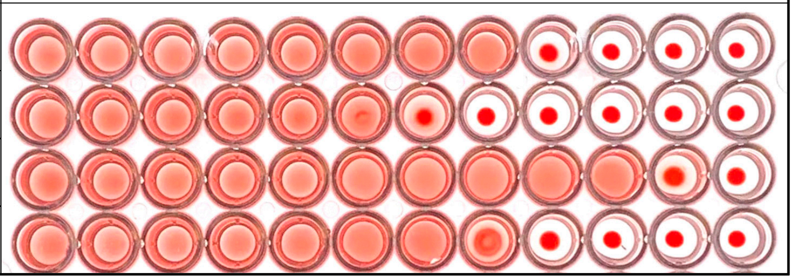

(**a**)

Row	PHA Isoform	Last + Well	HAUs	1	2	3	4	5	6	7	8	9	10	11	12
A	**PHA-P** 1 mg/mL by weight, **actual conc. 0.69 mg/mL (Sigma salt-free)** Serial dilutions starting at 1:1, A1-12	8	128												
B	**PHA-L Unconjugated. 1 mg/mL (Vector).** Serial dilutions starting at 1:1,B1-12	8	128												
C	**PHA-E + PHA-L Unconjugated. 1 mg/mL (Vector).** Serial dilutions starting at 1:1, C1-D12	12	2048												
D	PHA-E + PHA-L Unconjugated. 1 mg/ml (Vector). Cont.														
E	**PHA-E Unconjugated. 1 mg/mL (Vector).** Serial dilutions starting at 1:1, E1-F12	13	4096												
F	PHA-E Unconjugated. 1 mg/ml (Vector). Cont.														
G	**PHA-P Actual 1 mg/mL,** made 1.3mg/mL by weight (30% more PHA-P added) **(Sigma salt-free).** Serial dilutions starting at 1:1, G1-H12	10	512												
H	PHA-P Actual 1 mg/ml. Cont.														

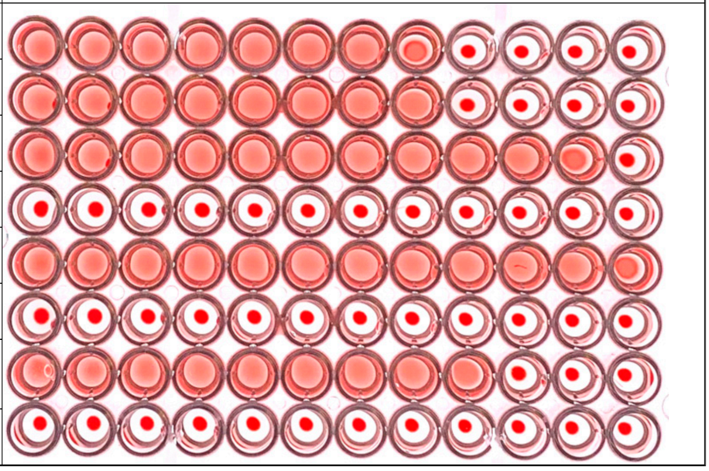

(**b**)

Figure S7. Hemagglutination of PHA isoforms: (**a**) Isoforms M, L, E and P from Millipore-Sigma; (**b**) Isoform P from Millipore-Sigma and isoforms L & E from Vector Laboratories.

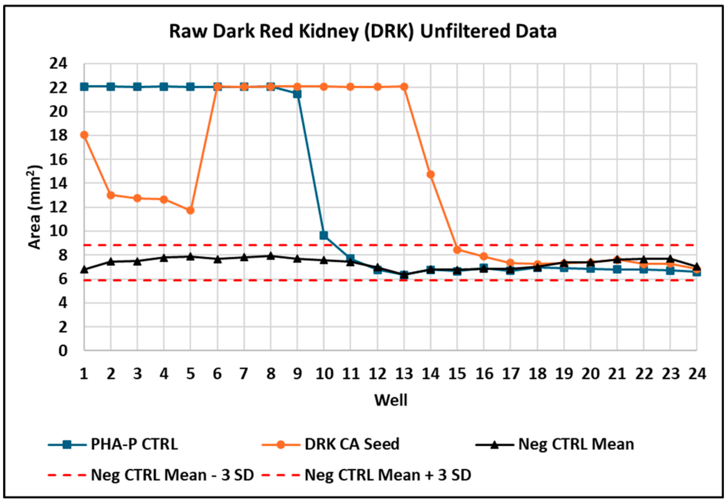

Figure S8. Image analysis profile of a commercially available raw DRK bean seed. Image-derived data analysis of hemagglutination results (original unfiltered data); hemagglutination area (mm^2) values > 3 SD above the mean negative control indicate active lectin in a well; CA: commercially available product; DRK: dark red kidney; HAU: hemagglutination unit; IA: image analysis; Neg CTRL: negative control; PHA-P CTRL: phytohemagglutinin isoform P positive control.

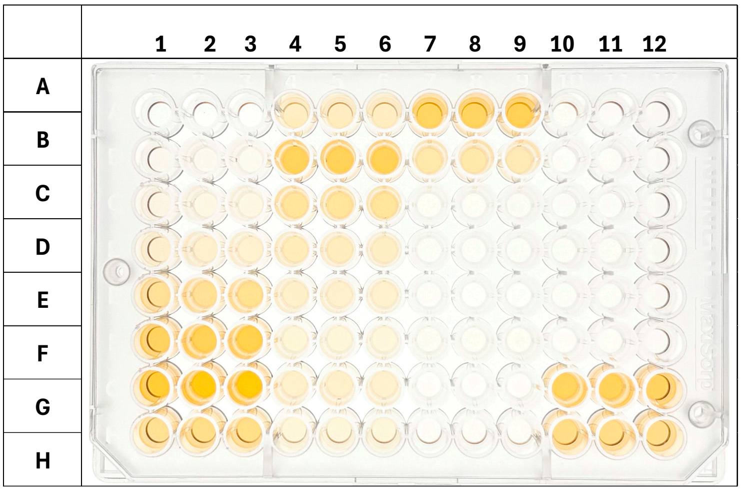

Figure S9. Representative ELISA plate. PHA-P standards run in triplicate (wells 1-3) are shown increasing in concentration ranging from 12.5 – 399.36 ng/100 µL (rows B-G) with a blank loaded in row A (wells 1-3).

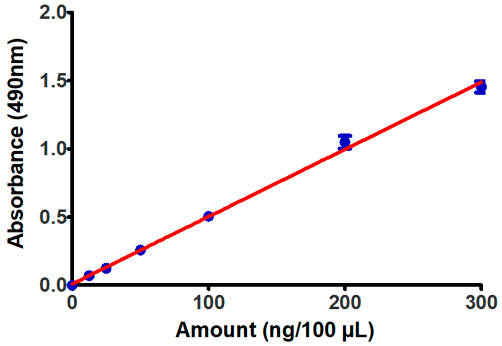

Figure S10. PHA-P ELISA standard curve.

Reference tables for computing amounts

Below are three reference tables for the hemagglutination assay, one for PHA-P (Table 2), and two for unknown samples (Tables 3 and 4), showing the respective dilution for each well, corresponding HAU, concentration, and the amount (ng). For unknown samples, when making extracts at a 10% w/v, in addition to the respective dilution and HAU for each well it includes the amount of sample represented in each well in mg and ng. These tables can be used to quickly calculate the amount of PHA-P in unknown samples.

Table S2. Reference Table for PHA-P Positive Control in the Hemagglutination Assay

Well	1	2	3	4	5	6	7	8	9	10	11	12
Dilution	1:1	1:2	1:4	1:8	1:16	1:32	1:64	1:128	1:256	1:512	1:1024	1:2048
HAU	1	2	4	8	16	32	64	128	256	512	1024	2048
Conc. of PHA-P (mg/mL)	1.04	0.52	0.26	0.13	0.065	0.0325	0.01625	0.00813	0.00406	0.00203	0.00102	0.00051
PHA-P in the well (ng)	104,000	52,000	26,000	13,000	6,500	3,250	1,625	812.5	406.25	203.13	101.56	50.78

Table S3. Reference for Unknown Samples in the Hemagglutination Assay

Well	1	2	3	4	5	6	7	8	9	10	11	12
Dilution	1:1	1:2	1:4	1:8	1:16	1:32	1:64	1:128	1:256	1:512	1:1024	1:2048
HAU	1	2	4	8	16	32	64	128	256	512	1024	2048
Amt. in well (mg)	10	5	2.5	1.25	0.625	0.313	0.156	0.078	0.039	0.020	0.010	0.005
Amt. in well (ng)	10,000,0000	5,000,000	2,500,000	1,250,000	625,000	312,500	156,250	78,125	39,062.5	19,531.3	9,765.6	4,882.8

Table S4. Reference for Unknown Samples in the Hemagglutination Assay

Well	13	14	15	16	17
Dilution	1:4096	1:8192	1:16384	1:32768	1:65536
HAU	4096	8192	16384	32768	65536
Amount of unknown powder in the well (mg)	0.00244	0.00122	0.00061	0.000305	0.000153
Amount of unknown powder in the well (ng)	2,441.4	1,220.7	610.3	305.2	152.6

Wells 18-24 are not shown for simplicity.

Table S5. Hemagglutination assay limitations

Section	Key Concepts	Summary
Definition of HAU	Semiquantitative measure of RBC agglutination	HAU indicates a protein's ability to agglutinate RBCs; 1 HAU = smallest amount of protein that results in agglutination.
Chemical & Molecular Basis	Protein-RBC interactions	HA proteins bind sialic acid receptors on erythrocytes
Erythrocyte Factors	Species-specific receptor profiles	Agglutination depends on RBC species; enzyme treatment; mechanical defibrination, can enhance or modify hemagglutination.
Reaction Conditions (Buffers & Reagents)	pH, ionic strength, and temperature	Optimal pH and ionic strength vary by protein; temperature affects cross-linking stability.
Mathematical Basis of HAU	Serial dilution and endpoint	HAU is determined by twofold serial dilutions; endpoint = highest dilution causing agglutination; titer = reciprocal of dilution (e.g., 1:64 = 64 HAU).
Sources of Variability	Dilution error, carryover, reagent variation, physical conditions	Pipetting inaccuracies, reagent inconsistencies, microplate materials, and vibration can distort results. Standardized reagents and physical conditions minimize bias.
Interpretation	Subjective endpoint reading and dilution error	Visual assessment introduces bias; discrete dilutions create measurement uncertainty. Image analysis is more objective when interpreting results.
Reproducibility	Inter-lab consistency	Standardized protocols, reagents, physical conditions, and training are crucial for consistency.
Comparative Assays	Comparison with other assays	HAU is a functional but relative unit; alternative quantitative assays, e.g., ELISA, offers high precision, but is more complex and time consuming.
Future Directions	Standardization & automation	Ongoing development of unified reagents and scoring algorithms using digital quantification tools to enhance precision and interlaboratory comparability.

ELISA, enzyme linked immunosorbent assay; HA, hemagglutination; HAU, hemagglutination unit; RBC, red blood cell.
